# Research progress on chemical metabolites, processing technologies, and pharmacological activities of asperosaponin VI: a systematic review and critical evaluation

**DOI:** 10.3389/fphar.2026.1725604

**Published:** 2026-04-10

**Authors:** Shitao Zou, Zhiwen Hou, Haibo He, Lina Zou, Huaqing Lin, Haiming Tang, Shimei Li, Jie Xu, Jihong Zhang, Hongwu Wang

**Affiliations:** 1 Hubei Key Laboratory of Natural Products Research and Development and Yichang Key Laboratory of Development and Utilization of Health Products with Drug and Food Homology, China Three Gorges University, Yichang, China; 2 Hubei Hengen Fulin Pharmaceutical Co., Ltd., Yichang, China; 3 College of Food Science and Technology, Huazhong Agricultural University, Wuhan, China; 4 Traditional Chinese Medicine Hospital of China Three Gorges University and Hubei Clinical Research Center for Functional Digestive Diseases of Traditional Chinese Medicine, Yichang, China; 5 Department and Institute of Infectious Disease, Tongji Hospital, Tongji Medical College, Hua Zhong University of Science and Technology, Wuhan, China

**Keywords:** asperosaponin VI, chemical metabolites, *Dipsacus asper*, pharmaceutical processing, pharmacological effects, systematic review

## Abstract

The purpose of this review is to systematically categorize and critically evaluate the chemical metabolites, processing methods, and pharmacological effects of the core active ingredient asperosaponin VI (ASD VI) sourced from *Dipsacus asper*. By following the principles of systematic review and best practices of ethnopharmacology, we analyzed available literature up to the year 2025. The results indicate that more than one hundred metabolites have been identified and reported in *D. asper*, including triterpenoid saponins, iridoids, phenolic acids, and alkaloids, among which ASD VI is the main active marker. Traditional wine-frying, salt-frying, and sweating processes have been known to significantly elevate the content and dissolution rate of ASD VI through biological transformations or physical and chemical changes, which enhance its effects of strengthening bones, tonifying the kidney, and hemostasis. ASD VI itself exhibits multiple pharmacological activities, such as promoting bone formation, offering neuroprotection, improving metabolic liver disease, enhancing myocardial protection, and preventing miscarriage. Its roles involve regulation of key signaling pathways like BMP/Smad, Wnt/*β*-catenin, and PI3K/AKT. However, our critical analysis reveals that current research efforts have some common limitations like the use of single model approaches, mechanisms mostly being evaluated at the correlation level, and missing data regarding the critical pharmacokinetics and clinical transformations. As a pilot effort, we systematically review the chemical composition, processing modifications, and component pharmacology of *D. asperoides* within a common framework that not only integrates existing knowledge but also reveals the core scientific bottlenecks from traditional experience to modern drug development; this is expected to provide a clear roadmap for in-depth research and development of ASD VI in the future.

## 1 Introduction

*Dipsacus asper* is a medicinal plant belonging to the same family as *Sambucus chinensis* (phylum Streptophyta, class Equisetopsida, subclass Magnoliidae) and is known to grow natively in central and southeast Asia. The medicinal extracts of this plant are derived from the dry roots of *D. asper* Wall. ex DC. (Dipsacus asper Wall. ex DC. | Plants of the World Online | Kew Science), which is a perennial plant that is primarily distributed in temperate biota. In traditional Chinese medicine (TCM), the extract of this plant is described as being bitter or acrid in taste, with a tepid constitution that is distributed to the liver or kidney channel; it serves the purpose of “nourishing the liver and kidney, strengthening the muscles and bones, continuing the healing of fractures, and stopping of disintegration and leakage.” Clinically, the extract has been mainly used to treat conditions like deficiencies of the liver and kidney, soreness and weakness of the waist and knees, rheumatoid arthritis, traumatic injuries, tendon injury fractures, metrorrhagia, metrostaxis, and threatened abortions (Chinese Pharmacopoeia Commission, 2025). The earliest records detailing the use of *D. asper* extracts date back to approximately 200 BC, and its Chinese name reflects its ability to “break and continue” (Su, 1981). *D. asper* boasts a lengthy legacy of medical use in China; although the main extract of the plant has been historically derived from *D. asper*, there are complexities regarding its origin and varieties. The primary components are also known to be sourced from legumes, plants of the Liliaceae family, mulberry, elderberry, thistle, and wild sesame; later on, the main sources included stone dragon grass and *D. asper* intermittent. In modern times, there have been over 20 potential documented sources for the medicinal extracts, including *D. japonicus*, Tibetan teasel, and *D. fullonum*, which reportedly grow in places like Russia, Kazakhstan, Uzbekistan, and west Asia (Dong, 2007; Editorial Committee of Flora of China, Chinese Academy of Sciences, 1986). At present, *D. asper* Wall. ex Henry is officially recognized as the primary botanical source for medicinal *D. asper* (Chinese Pharmacopoeia Commission, 2025). *D. asper* interrupts mainly grow in humid mountainous areas and are widely distributed in Sichuan, Hubei, Yunnan, Guizhou, and other provinces in China; among these, Sichuan is the largest producer while Hefeng and Wufeng counties in Hubei produce the highest-quality *D. asper*, which is traditionally known as “Wuhe Dipsacus” (Zhang, 2003).

In recent years, many scholars have isolated more than 100 metabolites from *D. asper*, including triterpenoid saponins, iridoid glycosides, phenolic acids, alkaloids, lignans, and essential oils (Tao et al., 2016). Among these, asperosaponin VI (ASD VI) sourced from the Dipsacus radix is the primary active ingredient in asperginin, which has pharmacological effects like protection of bone tissues (Li et al., 2022), prevention of recurrent spontaneous abortion (RSA) (Gao et al., 2010), analgesic and anti-inflammatory properties (Lu et al., 2013), as well as protection of nerves (Zhou et al., 2009), hepatic system (Li et al., 2014a), and cardiomyocytes (Li et al., 2010). Although there have been increasing research studies on this component, current knowledge remains fragmented as extant reviews mostly focus on the complete plant and lack a systematic summary of ASD VI; most studies also provide only activity descriptions and pathway preliminary reports without critical reviews of the model limitations, data consistency, and causal mechanisms. Although there has been partial scientific confirmation regarding the processing technology affecting the efficacy, the underlying mechanisms of chemical composition transformation and selective regulation of efficacy remain unclear, representing a “black box” between traditional experience and modern pharmacology.

To address these gaps, we aimed to overcome the limitations of traditional descriptive reviews and carry out a systematic integration and critical evaluation of the knowledge regarding ASD VI. The novelty of this review is mainly reflected in the following three aspects. First, we systematically review this iconic component to make up for the shortcomings of extant reviews that are mostly focused on the whole plant level. Second, we integrate the chemical properties of this component with the dynamic changes caused by processing and multisystem pharmacological mechanisms to construct an overall cognitive framework of “structure-processing-function” and overcome the “black box” connecting traditional processing experience and modern pharmacology. Third and most importantly, we critically consider the methodological rigor; here, we strictly follow the framework of ethnopharmacology best practices (four pillars) through a preregistered search strategy, transparent Preferred Reporting Items for Systematic reviews and Meta-Analyses (PRISMA) screening process, and structured quality assessment of all the original studies included. This ensures that our review is not only a summary of existing literature but also an objective assessment of the strengths of available evidence, research weaknesses, and future priorities. Through our approach, we aim to develop an in-depth method involving empirical description, mechanism interpretation, and clinical transformation.

## 2 Methodology

To ensure the systematicity, transparency, and repeatability of our review, we developed a detailed literature search, screening, and data synthesis strategy based on the core principles of the PRISMA guidelines and best practices of ethnopharmacology (four pillars).

### 2.1 Literature retrieval strategy

We conducted a systematic search of Chinese and English literature published up to 1 December 2025. The search databases included PubMed, Web of Science, Scopus, ScienceDirect, the Chinese database CNKI, Wanfang Data knowledge service platform, and VIP Chinese periodical service platform.

The search keywords included combinations with subject words and free text terms. The English literature search formula was as follows: (“Dipsacus asper” OR “Xu Duan”) AND (“Asperosaponin VI” OR “Akebia saponin D” OR “*Dipsacus asper* saponin VI”) AND (“chemical” OR “constituent” OR “processing” OR “processed” OR “pharmacology” OR “mechanism” OR “pharmacokinetic”). The search formula for the Chinese literature included the corresponding Chinese keywords. The retrieval process did not have an initial limit on the language, and the references of the obtained literature were screened retrospectively to avoid omissions.

### 2.2 Literature inclusion and exclusion criteria

The literature inclusion criteria were as follows: ASD VI isolated or identified from *D. asper* Wall. ex DC. was defined as the study object; the research content involved the chemical structure identification, processing technologies, pharmacological activities, or action mechanisms of ASD VI; experimental study of the original data (including *in vitro* and *in vivo* experiments).

The literature exclusion criteria were as follows: the studies only investigated the crude extract of *D. asper* and did not specifically analyze ASD VI; reviews, comments, conference abstracts, and dissertations (to maintain consistency of evidence, except for discoveries and important dissertations as cited in the discussion section); unobtainable full-text information or data published repeatedly.

### 2.3 Literature screening and data extraction

**FIGURE 1:**
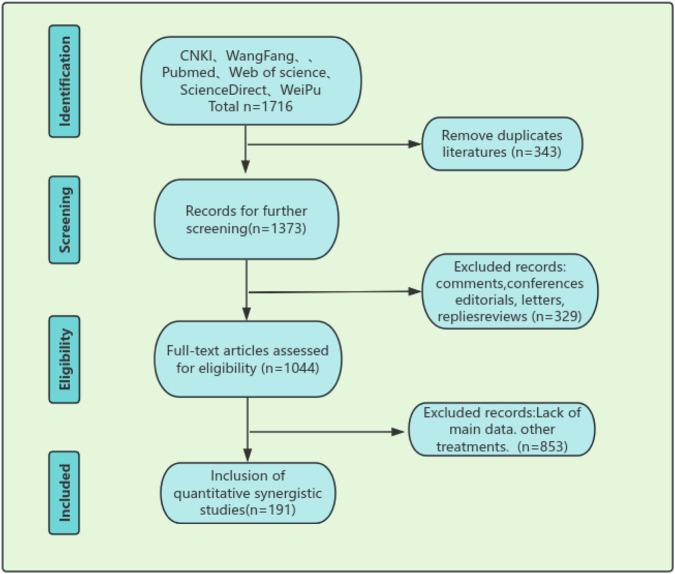
PRISMA flowchart.

All retrieved literary works were independently managed by two researchers. Initially, one of the researchers used a document management software (NoteExpress) to remove duplicate records. Subsequently, a preliminary screening was conducted by reading the titles and abstracts to exclude significantly unrelated studies. The full texts of the works retained at the end of preliminary screening were obtained, and these full texts were reviewed by two researchers independently in accordance with the inclusion and exclusion criteria. Any disagreement was resolved through discussion or consultation with a third researcher. The final results from the screening process are depicted in the PRISMA flowchart of Figure 1.

We extracted the following data from the selected literature using a preset standardized table: first author, year of publication, type of study (chemical/processing/pharmacology), experimental model/system, dosage or concentration of ASD VI, main research results, signaling pathways or mechanisms involved, and research conclusions. The data extraction was also completed by two researchers independently and cross-checked.

### 2.4 Evidence synthesis and quality assessment methods

Given the high heterogeneity among the models, methods, and endpoints of the studies included in this work, a quantitative meta-analysis is not feasible. Therefore, we use a narrative synthesis method to classify, compare, and summarize the evidence according to themes (chemistry, processing, and pharmacology).

To ensure rigorous evaluations of the original research, we conducted a critical quality assessment of all included pharmacological studies based on the framework provided in the best practice guidelines for ethnopharmacology. The specific assessment involved filling out the good practice (GA) forms (1 and 2a) covering the following key dimensions:

1. Plant material quality: species identification, sources, extract standardization information
2. Rigorousness of pharmacological experiment design: model correlation, sample size, control group setting (positive/negative control), dose selection basis, and appropriateness of statistical analysis method
3. Depth of mechanism research: whether the research involves a phenotypic description or molecular target verification
4. Interpretability and limitations of data: whether the results are clearly presented and study limitations are discussed.

The completed GA form is available as Supplementary File S1, which provides a structured evidence basis for the critical analysis in this review.

### 2.5 Declaration

1. All studies included in this review declared in their original texts that they met all necessary ethical requirements. The present review did not involve experiments with animal or human subjects.
2. The chemical characterizations and standardization of the extracts or compounds declared in the studies included herein have been explained in their original texts and will not be repeated in this review.

## 3 Chemical metabolites of *D. asper*

**FIGURE 2:**
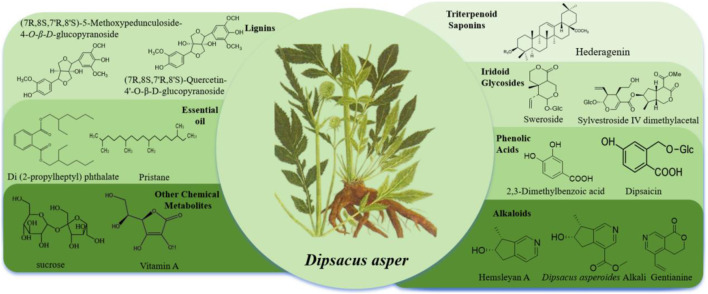
Classification of specific compound classes in *Dipsacus asper* and their representative metabolites.

As an herb used in TCM, *D. asper* exhibits a diverse and complex chemical composition that underpins its pharmacological activities. In recent years, advances in separation and identification technologies have enabled the systematic identification of multiple classes of metabolites in *D. asper*, including triterpenoid saponins, iridoids, phenolic acids, alkaloids, lignans, and essential oils. Among these, ASD VI stands out as a representative bioactive marker compound. Its structural features, biotransformation pathways, and correlation with therapeutic efficacy have become the focus of key research efforts. In this section, we provide a detailed overview of the chemical constituents reported in *D. asper* as well as offer a chemical basis for subsequent studies on quality control, processing mechanisms, and pharmacodynamic material foundations. Figure 2 provides a visual summary of the specific chemical compound classes identified in *D. asper*.

### 3.1 Triterpenoid saponins

The phytochemical studies showed that the chemical metabolites of *D. asper* are mainly triterpenoid saponins. To date, researchers have isolated numerous triterpenoid saponins through different extraction, separation, purification, and appraisal processes from a total of 33 species, of which 31 have the same skeleton (as shown in Supplementary Figure S1). The characteristics of triterpenoid saponins are as follows: the sugar chain is mainly composed of pyranoarabinose, glucose, rhamnose, and xylose and that C-28 is often combined with one or two glucose-forming ester glycoside bonds or free COOH; C-3 is an oleanane-type triterpene with a pentacyclic system that connects straight or branched sugar chains composed of 1–6 monosaccharides by an alcohol-glycoside bond. The chain sequence is arabinose, rhamnose, glucose, glucose, and xylose from the inside to the outside, and the branched chain is usually a rhamnose-connected third sugar (glucose). When their aglycones are replaced by sugar groups at positions 3 or (and) 28 (i.e., R1 and R3, as shown in Supplementary Figure S1), they are oleanane-type, while the linked hydroxyl group at position 23 is an ivy saponin (Chang et al., 2019).

Since Wei and Liang (Wei Huifen and Liang Guangyi, 1987) extracted Hederagenin (1) come from the root of Dipsacus asper in 1987, scholars began to study the chemical metabolites of Dipsacus asper. (Zhang Yongwen and Xiu Zhi, 1991a; Zhang Yongwen and Xiu Zhi, 1991b; Zhang Yongwen and Xiu Zhi, 1992; Zhang Yongwen and Xiu Zhi, 1993) isolated Dipsacus saponin IV (2), Dipsacus saponin V (3), Dipsacus saponin VI (4), Daucosterol (32), Macranthoidin A (5), Dipsacus saponin VIII (6), Dipsacus saponin IX (7), Dipsacus saponin X (8), Dipsacus saponin XI (9), Dipacus saponin XII (10) and Dipsacus saponin XIII (11); Yang Shangjun et al. 1993 isolated Dipsacus saponin A (12), Cauloside A (13), HN saponin F (14) and 3-O-[β-D-xylopyranosyl (1→4)-β-D-glucopyranosy1 (1→4)] [α-L-rhamnopyranosyl (1→3)]-β-D-glucopyranosyl (1→3)-α-L-rhamnopyranosyl (1→2)-α-L-arabinopyranosyl-hederagenin-28-O-β-D-glucopyrano side (15); Jung et al. 1993 isolated Dipsacus saponin B (16), Dipsacus saponin C (17), Macranthoside B (18) and 3-O-β-D-xylopyranosyl (1→4)-β-D-glucopyranosyl (1→4)-β-D-glucopyranosy (1→3)-α-L-rhamnopyranosyl (1→2)-α-L-arabinopyranosyl-hederagenin (19); Wei Feng et al. 1994 isolated Dipsacus saponin F (20) and Dipsacus saponin H1 (21); Oh et al. 1999 and (Liao Zhenchun et al., 1999) isolated 3-O-β-D-glucopyranosyl (1→3)-α-Lrhamnopyranosyl (1→2)-β-L-arabinopyranosyl-hederagenin-28-O-β-D-glucopyranosyl 1→6)-β-D-glucopyranoside (22), 3-O-β-D-glucopyranosyl (1→3) [α-L-rhamnopyranosy1(1→2)]α-L-arabinopyranosyl- hederagenin-28-O-β-D-glucopyranosyl (1→6)-β-D-glucopyranoside (23), 3-O-α-L-rhamnopyranosy (1→3)-β-D-glucopyranosyl (1→3)-α-L-rhamnopyranosyl (1→2)-α-L-arabinopyranosyl-hederagenin (24) and 3-10 O-β-D-glucopyranosyl (1→4)[α-L-rhamnopyranosy1 (1→3)]-β-D-glucopyranosyl (1→3)-α-L- rhamnopyranosyl (1→2)-β-L-arabinopyranosyl-hederagenin-28-O-β-D-glucopyranosyl (1→6)-β-D-glucopyranoside (25); Hung et al. 2005 isolated Kalopanaxsaponin A (26), 3-O-β-D-xylopyranosyl(1→3)-α-L-rhamnopyranosyl (1→2)-α-L-arabinopyranosyl-hederagenin (27) and 3-O-β-D-glucopyranosyl (1→3)-α-L-rhamnopyranosyl (1→2)-α-L-arabinopyranosyl-hederagenin (28); Wang Jianping et al. 2006, Tian et al. 2007 identified ursolic acid (33) and oleanolic acid (29); Liu et al. 2011 identified Dipsacus saponin J (30) and Dipsacus saponin K (31) (as shown in Supporting Information).

At present, the extraction of triterpenoid saponins and other chemical metabolites from natural plants is usually achieved by the ultrasonic or reflux method. One study demonstrated that the transfer rate did not indicate significant variation of saponin extraction from the ultrasonic to reflux methods (Zhu et al., 2014). Furthermore, the extraction study indicated that the saponin composition of *D. asper* sourced from different regions varied to some extent. For example, the total saponin contents of *D. japonicus* and *D. fullonum* were low, while *D. asper* saponin XII was the main content without ASD VI and *D. asper* saponin X. ASD VI and *D. asper* saponin X were the main metabolites of *D. asper* produced in China, whereas *D. asper* saponin XII had a low content. It is anticipated that advancements in analytical technologies would enable discovery of more metabolites and differences in the Dipsacus Linn. to better distinguish authentic medicinal botanical drugs from counterfeit drugs while promoting the development of the Chinese medicine market.

### 3.2 Iridoid glycosides

At present, scholars have isolated 21 kinds of iridoid terpenoids from *D. asper* plants, which can be divided into simple and polymeric iridoid terpenoids according to the parent nucleus, with the polymeric iridoid terpenoids being further divided into dimers and tetramers. The first isolates reported were sweroside (34), loganin (35), and cantleyoside (36) by Kauno et al. (1990). Subsequently, Tomita and Mouri (1996), Wei and Lou (1996), and Tomassini et al. (2004) successively isolated 6′-*O*-*β*-*D*-glucopyranosylloganin (37), loganic acid (38), 6′-*O*-*β*-*D*-glucopyranosylloganic acid (39), sylvestroside III (40), and sylvestroside IV dimethylacetal (41). Tian et al. (2006, 2007) isolated dipsanoside A (42), dipsanoside B (43), dipsanoside H (44), 6′-*O*-*β*-*D*-apiofuranosyl sweroside (45), dipsanoside C (46), dipsanoside D (47), dipsanoside E (48), dipsanoside F (49), triplostoside A (50), dipsanoside G (51), and lisianthioside (52). More recently, Sun et al. (2017) isolated two new metabolites, namely, dipsanoside M (53) and dipsanoside N (54) (Supplementary Table S2; Supplementary Figure S2).

### 3.3 Phenolic acids

At present, 16 phenolic acid metabolites have been identified from plants of the *D. asper* genus. Abdallah (1991) first isolated dipsaicin (55) from *D. asper*, while Kim et al. (1999) isolated protocatechuic acid (56) and caffeic acid (57). Cao et al. (2010), Hung et al. (2018), Tian et al. (2007), Wang et al. (2014), and Tao et al. (2016) isolated 3,4-di-*O*-caffeoylquinic acid (58), methyl 3,4-di-*O*-caffeoylquinate (59), 3,5-di-*O*-caffeoylquinic acid (60), methyl 3,5-di-*O*-caffeoylquinate (61), 4,5-di-*O*-caffeoylquinic acid (62), methyl 4,5-di-*O*-caffeoylquinate (63), 2,6-dihydroxycinnamic acid (64), vanillic acid (65), 2′-*O*-caffeoyl-*D*-glucopyranoside ester (66), caffeoylquinic acid (67), chlorogenic acid (68), 5-caffeoylquinic acid (69), and 4-caffeoylquinic acid (70). These 16 phenolic acids are common to many plants (Supplementary Table S3; Supplementary Figure S3).

### 3.4 Alkaloids

The alkaloids contained in *D. asper* are mainly camptothecin (71), *D. asper* base (72), and gentianine (73). Although some studies reported that only camptothecin and *D. asper* base are contained in *D. asper*, it is clear that gentianine is converted from other metabolites during the extraction process (Ding et al., 2005a; Yang et al., 1993a, b; Yang et al., 1996) (Supplementary Figure S4).

### 3.5 Lignins

Sun et al. (2014) isolated (7R, 8S, 7′R, 8’s)-5-methoxyapigenin-4-*O-β-D*-glucopyranoside (74), (7R, 8S, 7′R, 8’s)-apigenin-4-*β*-d-glucopyranoside (75), and acanthoside *D* (76) using macroporous resin, reversed phase silica gel, and preparative high-performance liquid chromatography (HPLC), along with (7R, 8S, 7′8′R, S)-quercetin-4′-*O*-beta-*D*-glucose pyranoside (77), (7R, 8S, 7′8′R, S)-8-hydroxy benzene and stilbene cinnamyl-4′-*O*-beta-*D*-grapes pyranoside (78), and (7R, 8S, 7′8′R, S)-8-hydroxy benzene and stilbene cinnamyl-4-*O*-pyran beta-*D*-grapes glucoside (79). These metabolites are ubiquitous in medicinal botanical drugs and exhibit multiple bioactivities like antioxidant, anti-inflammatory, anticancer, antiviral, and antidiabetic properties, thereby holding significant value in pharmacological and phytochemical research (Supplementary Figure S5).

### 3.6 Essential oils

Zhang et al. (2024) used gas chromatography mass spectrometry (GC-MS) to identify 51 volatile oils from *D. asper*, where two of them remain uncharacterized. The volatile oil of *D. asper* contains more phenols, fewer terpenoids, the largest relative content of alcohols, and the largest types of hydrocarbons. The chemical component with the largest relative content is γ-sitosterol (35.80%), followed by soybean sterol (8.37%), 24-methyl-5-cholestene-3-ol (7.06%), and lanosterol (6.71%) (Supplementary Table S4).

### 3.7 Other chemical metabolites

In addition to the above metabolites, there are many common metabolites in *D. asper*, such as polysaccharides, sucrose, vitamins, and inorganic elements like calcium, iron, magnesium, zinc, and copper. Notably, there is a high concentration of the trace element titanium (Ding et al., 2005b; Yang et al., 1996).

## 4 Pharmaceutical processing of *D. asper*

The TCM preparations embody ancient pharmaceutical wisdom for preparing decoction pieces. There are many processing methods of *D. asper*, including cleaning, cutting, frying, and wine treatment, and the commonly used processing methods in modern times are sweating, wine stir-frying, and saltwater stir-frying (Jin et al., 2010). As ASD VI is the primary bioactive constituent in *D. asper*, the processing of *D. asper* is based on the content comparison standard to judge the rationality of processing (Liu et al., 2009). The 2025 edition of the Chinese Pharmacopoeia only includes three kinds of clinical applications and standards, namely, raw (Sichuan intermittent), wine stir-frying, and salt stir-frying, all of which entail ASD VI content as the quality standard and stipulate that it should be ≥1.5% (Chinese Pharmacopoeia Commission, 2025). Other relevant studies on *D. asper* mostly used ASD VI as the content determination index (Tan et al., 2006; Wang and Wang, 1991; Zhang et al., 2012a). Given the differences in the natural environments of different regions, the average ASD VI content varies in the raw *D. asper* product from different regions (Supplementary Figure S7). Therefore, the impacts of processing on *D. asper* are evaluated on the basis of percentage of content change in subsequent comparisons (Cao G. et al., 2011; Du et al., 2013; Guo et al., 2010; Peng et al., 2015; Yang et al., 2011; Zhang Q. J. Y. et al., 2020a; Zhou T. M. et al., 2021; Luo et al., 2015).

### 4.1 Sweating

“Sweating” is an important processing method in Chinese medicine, which can promote the internal water distribution of medicinal materials and speed up drying (Duan et al., 2013); furthermore, it regulates and promotes the vitality of the enzyme system and microbial community in the tissues of medicinal materials, while initiating or accelerating the biological transformations and chemical conversion processes of essential/specialized metabolites, thereby changing the medicinal properties of natural medicinal substances and directly affecting the phytopharmaceutical quality (Yang et al., 2000). Hence, “sweating” reflects the true, false, superior, and inferior characteristics of Chinese medicinal materials in terms of appearance, color, aroma, and taste (Chen and Xu, 2014).

It is noted that the appearance of *D. asper* after “sweating” is bean green, dark green, or tan, while traditional beliefs indicate that dark green *D. asper* sections are the best products after “sweating” (Jiangxi Provincial Drug Administration, Department of Health, 1991; Ministry of Health of the Peopleʼs Republic of China, Drug Administration Bureau, 1988) (Supplementary Figure S6). Zhou T. M. et al. (2021), Zhou et al. (2022), and He et al. (2021) showed that sweating of *D. asper* affects the fungal community abundance in the medicinal materials and that the dominant bacteria that are enriched after sweating could promote the accumulation and color changes of ASD VI. Zhou T. M. et al. (2021) and Du et al. (2013) discovered that the composition of ASD VI was elevated subsequent to sweating of *D. asper* (Supplementary Figure S7), indicating that the sweating processing of *D. asper* may be associated with upregulated expressions of CYP450 and UGT as well as the downstream modification enzymes of the *D. asper* triterpenoid saponin biosynthesis pathway (He et al., 2021). Research on TCM shows that proper sweating can reduce the toxicity, change the pharmacological properties, retain the active metabolites, improve the taste or remove odors, and enhance the purity of TCM. However, the precise mechanisms underlying sweating remain to be elucidated as it is impossible to establish a reasonable modern “sweating” method; nonetheless, the TCM “sweating” techniques should be adhered to (Cao G. et al., 2011; Chen et al., 2018; Du et al., 2013; Du et al., 2014; Jin QI et al., 2011).

Modern research suggests that the efficiency of sweating may be a multifactor-driven biotransformation process. First, the hygrothermal environment inhibits primary metabolism and may flow the metabolism to the biosynthesis of secondary metabolites (such as saponins). Second, the succession of microbial communities is crucial; some dominant bacteria may directly secrete enzymes like *β*-glucosidase to catalyze the hydrolysis or transformation of saponin glycosyl groups to produce aglycones or secondary saponins with higher activities. Finally, the plant’s own stress responses are activated. Studies have shown that sweating can upregulate the expressions of key modification enzyme genes (such as CYP450s and UGTs) downstream of triterpene saponin biosynthesis in the roots of *D. asper*, which may be the direct molecular basis for the increase in ASD VI content. However, the above mechanisms need to be directly confirmed through sterile control experiments, metabolomics tracking, and transcriptomics analysis.

### 4.2 Stir-frying with wine

Stir-frying with wine has been in use since ancient times, and wine cooking is a widely utilized processing method in TCM (Xu et al., 2008). By mixing the medicine with a certain amount of wine and then frying, it is possible to change the medicinal properties, enhance promotion of blood circulation, correct odors, and eliminate fishiness. After processing with wine, the direction of action of the medicine changes to enhance efficacy; furthermore, the bioactive metabolites accumulated in hepatic and renal tissues upon processing with wine will enhance the nourishing effects (Tao et al., 2016). Accumulating evidence demonstrates that the analgesic, anti-inflammatory, and anticoagulant impacts of stir-frying with wine are far superior to those of crude drugs, and studies have shown that the regulatory capacity of stir-fried herbs with wine on the OPG/RANK/RANKL axis system in osteoporotic rats is stronger than that of crude drugs owing to enhancement of the pharmacological efficacy (Tao et al., 2016). Some scholars found that the content of ASD VI was upregulated after stir-frying with wine (Supplementary Figure S7) and that the bioavailability of ASD VI could be improved after stir-frying with wine (Luo et al., 2015; Lv et al., 2023; Peng et al., 2015; Tao et al., 2016; Zhang et al., 2010; Zhang et al., 2018; Zhang Q. J. Y. et al., 2020; Tao et al., 2016). At present, the specific reactions of wine processing are not clear, which may be the result of multiple factors; however, alcohol itself can excite the nervous system, and the conclusion that adding alcohol during drug processing can play a certain purpose in disease treatment is indisputable. Therefore, stir-frying with wine is still conducive to the application of *D. asper* in clinical treatment.

The mechanism by which wine enhances efficiency may go beyond simple dissolution and extraction. Ethanol as a reaction medium and reactant may be involved in three types of effects. In thermal catalysis, ethanol may promote the esterification or dehydration reactions of some hydroxyl groups in the saponin molecule during heating, slightly changing its polarity and thereby affecting its absorption and distribution. In the Maillard reaction potential, the Maillard reaction may occur between reducing sugars and amino acids in the medicinal materials under ethanol and heat application. The complex products may then interact with ASD VI or exert pharmacological activities alone to produce synergistic effects. In physiological modulation, ethanol may temporarily change the gastrointestinal environment or liver cell membrane fluidity, affecting the absorption and first-pass metabolism of ASD VI. Future research should thus focus on identifying the chemical derivatives produced upon wine processing as well as comparing the *in vivo* pharmacokinetic differences of the component groups before and after processing.

### 4.3 Stir-frying with saltwater

Stir-frying with saltwater is another TCM processing method, whose specific operation entails mixing the net medicinal materials or cut products with a certain amount of saltwater solution before heating in a pot or placing the net medicinal materials in a pot while stirring and spraying saltwater along with gentle heating, frying to a specified degree, and cooling (Zhang D. et al., 2012). Compared with stir-frying with wine, this procedure has a relatively late development history but is still based on the traditional theory that “salt draws drugs away from the kidney,” which is often used to promote the effects of drugs for renal function enhancement and diuresis. Meanwhile, studies have proven that *D. asper* enhances tonification of the liver and strengthens the kidney after salt processing (Wu Chuanjiang et al., 2018; Zhou Y. X. et al., 2021). Luo et al. (2015), Peng et al. (2015), Zhang Q. J. Y. et al. (2020), and Zhang et al. (2010) found that after *D. asper* was stir-fried with saltwater, the composition of ASD VI and its dissolution rate both increased (Supplementary Figure S7). Jin et al. (2013), Wu et al. (2023), and Zhang D. et al. (2012) summarized the effects of substituting conditions for the content of ASD VI during stir-frying with saltwater and compared the differences in *D. asper* slices before and after stir-frying with saltwater. After stirring and frying with salt, the strained *D. asper* is brownish yellow or dark brown in color while the taste is slightly bitter and salty. Gao et al. (2010) found that saponins and iridoid terpenoids increased significantly after intermittent salt burning, which may be the reason why discontinuous salt burning significantly enhanced tonicity of the kidney and strengthened the waist with downward movement of the drugs. These studies provide a more scientific theoretical basis for salt processing of the herbs.

The efficacy of salt-processed “downward drug introduction” (toward the kidney) has potential physical, chemical, and biological basis. The core features include ion effects as follows. Salting-out: The addition of sodium chloride changes the ionic strength of the extraction solvent and affects the solubility of saponins (salting-out effect), thereby changing the dissolution behaviors during decoction. Osmotic damage: a hypertonic salt solution can destroy the structure of the plant cell membrane, so that the active metabolites in the cell are released more easily. *In vivo* regulation: the ingested Na^+^ may create a more targeted microenvironment for ASD VI by affecting the ion exchange environment of the kidney or regulating the endocrine axis related to the kidney tonifying effect (such as the hypothalamus-pituitary-adrenal axis). This suggests that salt processing not only changes the metabolite content but also guides and synergizes drugs by regulating the internal environment of the body. These mechanisms need to be cross validated from the systemic and molecular perspectives.

### 4.4 Comprehensive comparison and research prospects of the processing mechanisms

Wine stir-frying, salt stir-frying, and sweating represent three different synergistic processing paths involving thermochemistry, physical ions, and biotransformation, respectively. The core of wine roasting is the synergy of heat and ethanol, which can lead to chemical transformations such as the Maillard reaction as well as change the dissolution and absorption behaviors of metabolites. Salt stir-frying mainly changes the dissolution of metabolites through ion effects (such as salting-out) and osmotic pressure damage, and the traditional drug-directing effect may be related to the introduction of Na^+^ to regulate the ion environment or drug distribution in the body. Sweating is a complex biological stress response process involving microbial-community-mediated transformations, endogenous enzyme activation, and upregulation of plant secondary metabolic pathways. However, the current understanding of the processing mechanism is mostly based on inference of the correlation between the metabolite content and efficacy changes before and after processing. There is still a lack of accurate identification of the key transformation products, direct causal proof of the physical and chemical biological events caused by processing and efficacy enhancement, and in-depth explanation on how processing systematically changes the *in vivo* processes of the metabolites.

To overcome these limitations, future research efforts must adopt a multidisciplinary strategy: first, chemical omics technology should be used to systematically identify characteristic markers and new metabolites produced during processing; second, it is necessary to integrate computational simulations and chemical biology methods to verify the direct targets of these transformation products. Furthermore, the *in vitro* microbial coculture or chemical-enzymatic hydrolysis model should be constructed to analyze the specific transformation pathways. It is particularly critical to carry out processing-oriented comparative pharmacokinetic studies to clarify the exposure differences of active metabolites in different processed products in the target tissues. Finally, based on the clarified mechanism, intelligent and monitored modern processing technologies can be developed. By realizing the leap from “experience description” to “mechanism interpretation,” we can not only consolidate the scientific connotations of processing *D. asper* but also provide a model for the modernization of TCM processing methods.

## 5 Pharmacological effects of *D. asper*

**FIGURE 3:**
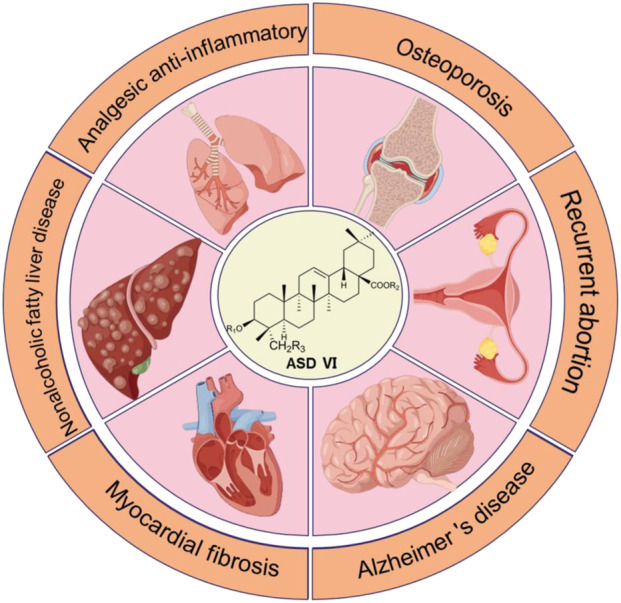
Pharmacological effects of asperosaponin VI (ASD VI).

The pharmacological effects of *D. asper* are very broad, and many ancient medical books have described these effects in great detail. However, the clinical applications of *D. asper* have changed over time. For example, *D. asper* was initially compatible with euperia, achyrania, citanola, mulberry parasite, etc. and was used to treat deficiencies of the liver and kidney or Yang deficiency of the kidney. Thereafter, it was used along with *Colla Corii Asini* (donkey-hide gelatin) and mugwort leaf to treat gynecological conditions, which may be related to its source changes. Several studies have reported that the primary active ingredient of *D. asper* is ASD VI (Dai et al., 2020; Yang et al., 2019), which is also known as saponin D or *D. asper* saponin C in literature; its molecular formula is C_47_H_76_O_18_, and it is a triterpenoid saponin compound in *D. asper* with great medicinal value. ASD VI exhibits a broad spectrum of pharmacological effects, such as promoting bone growth (Li et al., 2022), preventing RSA (Gao et al., 2010), analgesic and anti-inflammatory properties (Lu et al., 2013), and protection of the nerves (Li et al., 2013), liver (Wei et al., 2023), and cardiomyocytes (Li et al., 2010) (Figure 3).

### 5.1 Treatment of osteoporosis

Osteoporosis (OP) is a systemic skeletal disease caused by compromised bone microarchitecture, increased bone fragility, and elevated fracture risk. The basic pathological mechanism of OP is related to the weakened activity of osteoblasts (OBs) or increased activity of osteoclasts (OCs) in patients, which ultimately leads to loss of bone mass (Li et al., 2014). Current research on known biological process development of OBs is closely linked with the incidence of OP, and one of the important sources of OBs is bone marrow mesenchymal stem cells (BMSCs) as well as the related transcription factors and cytokines. BMSCs can further differentiate into OBs, thus promoting bone tissue formation and enhancing bone strength (Niu et al., 2011; Cai J. Q. et al., 2011). In recent years, many studies have shown that ASD VI is involved in the expressions of various signaling pathways, which can indirectly promote the maturation and differentiation of BMSCs and OBs while inhibiting OC formation, thus promoting bone tissue formation and playing an anti-OP role (Zhang Q. J. Y. et al., 2020; Ma et al., 2022; Zhang et al., 2012b; Niu et al., 2015; Liu, 2019). Zhang Z. D. et al. (2020) found that ASD VI (at concentrations of 0.1, 1, and 10 μM) promoted the synthesis of bone morphogenetic protein-2 (BMP-2) (Zhang et al., 2012a; Li et al., 2017) as well as activated the BMP/Smad signaling pathway by facilitating the activity of alkaline phosphatase (ALP) in the OBs (Zhang Z. D. et al., 2020). The combination of BMP-2 and BMP receptors can induce the formation of complex receptors as well as further phosphorylation of c-Jun and p38. The JNK signaling pathway in the mitogen-activated protein kinase (MAPK) signaling axis was successfully activated and phosphorylation of extracellular regulatory protein kinase (ERK) was promoted; the activation of ERK could inversely promote the expression of BMP-2 protein to promote maturation and distinction of OBs (Huang et al., 2018; Li et al., 2014b). BMP-2 can also promote the expression of the osteogenic gene *Runx2*, which then improves the activity of the phosphatidylinositol-3 kinase (PI3K)/protein kinase B (AKT) signaling pathway by increasing the PI3K and AKT protein levels to enhance the binding of *Runx2* to the corresponding transcribed DNA. The activity of *Runx2* was further enhanced during OB differentiation (Niu et al., 2011; Ke et al., 2016; Ghosh-Choudhury et al., 2002). In addition, ASD VI (at concentrations of 1, 10, and 50 μg/mL) was found to significantly enhance the regulation of the transcription and translation of osteogenic differentiation elements of BMSCs, such as ERK, p38, *β*-catenin, and Runx2, by interfering with the Wnt/*β*-catenin signaling pathway and upregulating OC differentiation factor induced receptor/osteoclast differentiation factor (OPG/RANKL). These inhibit the lipogenic differentiation of ST-2 cells and protect bone tissues (Zhang et al., 2012b; Wang et al., 2015; Zhang et al., 2022; Li et al., 2017). Other preliminary evidence suggests that the action mechanism of ASD VI (20, 40 mg/kg/d) on BMSCs may be related to activation of the semaphorin 3A pathway as well as expressions of Cbf*α*1 mRNA and other related substances (Wu et al., 2012; Li et al., 2021).

Although the above studies provide rich mechanistic clues regarding the anti-OP effects of ASD VI, there are several key limitations in the existing evidence system. First, at the model level, the studies are highly dependent on single OB cell lines (such as MC3T3-E1) and the ovariectomized rat model. Although the latter can simulate estrogen-deficient bone loss, it cannot fully represent the comprehensive pathology of an aging microenvironment, chronic inflammation, and vascular function decline, which are crucial in human senile OP. Second, there is significant heterogeneity in the effective dose/concentration range, and the effective *in vitro* concentration varies from micromolar to millimolar levels (Liu, 2019; Liu et al., 2020). These differences may be due to differences in the compound purity, cell model sensitivity, or detection endpoint, which make it difficult to determine a uniform biological activity threshold. Most importantly, the core premise of its clinical transformation and pharmacokinetic characteristics is completely unknown. The oral bioavailability, bone tissue targeting distribution ability, and *in vivo* metabolic pathway of ASD VI have not been elucidated, which prevents scientific deduction of the *in vivo* dosage from the effective *in vitro* concentration. Future studies need to prioritize systematic pharmacokinetic-pharmacodynamic studies and validate their efficacies in more complex osteoporosis models, such as the aging or glucocorticoid-induced OP models.

**FIGURE 4:**
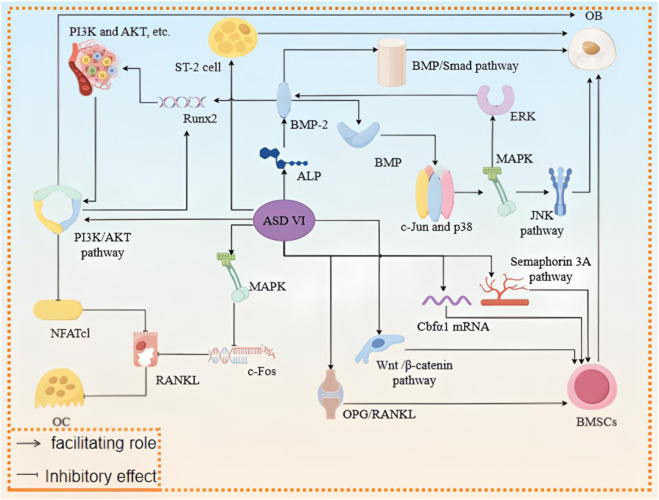
Mechanism of action of ASD VI in the treatment of osteoporosis.

Thus, ASD VI promotes osteogenic differentiation of BMSCs, promotes OB maturation, and inhibits OC formation by intervening in various signaling pathways to achieve bone tissue protection, showing good clinical efficacy against OP (Ding et al., 2019) (Figure 4) Detailed information on the above experiments as well as their advantages and disadvantages are summarized in Supplementary Table S5. However, the specific pathways are not clear and need further study.

### 5.2 Prevention of RSA

RSA is a clinically widespread gynecological condition that encompasses two or more unplanned abortions before 28 weeks of gestation (Zhang et al., 2022). Research has demonstrated that the occurrence of RSA may be linked to dysproliferation and excessive apoptosis of the villi and decidua cells as well as decreased blood progesterone levels during decidua necrosis in early pregnancy (Sun et al., 2017; Du et al., 2018). In clinical settings, TCM practitioners frequently consider *D. asper* as the first choice of treatment for RSA, and it has been proved that *D. asper* is closely associated with promoting expression of the progesterone receptor (PR). Du (2018) and Gao et al. (2010) used primary decidual and HeLa cell models and identified that ASD VI can activate PR advocate in both cell types, increase the expressions of the PR, activate the subsequent Notch signaling pathway, and trigger decidualization to dissect the implanted fertilized egg, thus helping to maintain pregnancy; although this provides a molecular mechanistic explanation for the traditional “tocolysis” effect, it should be noted that HeLa cells are a type of cervical cancer cell line and that their correlation to pregnancy biology research is limited; they are also known to have increased expressions of PSG1 and PR. Activation of the AKT signaling pathway upregulates Bcl-2 expression and downregulates Bax expression, which play pharmacological roles in preventing and controlling abortions. However, these mechanisms are currently mainly confirmed in cell models, so parallel verifications are still needed in animal abortion models.

The study of the role of ASD VI in an abortion model provides a modern biological explanation for understanding the traditional tocolysis effect. However, there are challenges to the transformation relevance of current evidence, with the main limitation being the single model approach. Extant works focus on cell models or animal abortion models with hormone interventions. These models are not sufficient for simulating the complex immunology, genetics, and prethrombotic state of human RSA. Next, the mechanistic research efforts are still in the pathway association stage, and the exact regulation of PR and its downstream signals (such as direct activation or transcriptional regulation) by ASD VI has not been confirmed at the molecular level. In addition, vital reproductive toxicology and pregnancy safety data are not available. Evaluating the effects of ASD VI on embryonic development, placental functions, and long-term health of the offspring is an absolute prerequisite for its use during pregnancy. In the future, it is necessary to verify the effects of ASD VI in models closer to the etiology of human RSA, such as autoimmune models, and strictly evaluate its pregnancy safety. Detailed information on the above works as well as the advantages and disadvantages are available in Supplementary Table S6.

### 5.3 Analgesic and anti-inflammatory properties

As a traditional wind-dampness medicine, *D. asper* has positive curative effects on rheumatism and arthralgia, fall-flutter damage, tendon damage, and bone breaks. In the mouse model of allergic airway inflammation, Xuan et al. (2024) found that treatment with ASD VI increased the expression of p-AMPK in the lung tissue, which has protective effects against allergic airway inflammation and anti-inflammatory effects via inducing AMPK activation. However, the specific activation of AMPK (directly or indirectly) remains to be elucidated. In the lipopolysaccharide (LPS)-stimulated macrophage model, Luo et al. (2023) identified that the anti-inflammatory effects of ASD VI could suppress M1-type polarization of the macrophages while facilitating M2-type polarization, and anti-inflammatory immune regulation is achieved by mitigating the polarization of M1/M2-type phagocytes. However, this acute strong inflammatory stimulation model is different from the pathological background of chronic inflammation. Luo et al. (2023) and Gong et al. (2019) showed that ASD VI offered anti-inflammatory protection by inhibiting the activation of the interleukin-6 (IL-6)/STAT3 pathway, downregulating the expression of DNMT3b, regulating the polarization of macrophages, and activating LPS to stimulate the nuclear factor erythroid-2-related factor 2 (Nrf2) pathway in macrophages, thereby reducing the occurrence of inflammatory responses. Some scholars have studied the analgesic mechanisms and found that ASD VI may be linked to the improvement of the expressions of GABA receptors and adrenergic receptors (Suh et al., 1996).

Studies on the anti-inflammatory and analgesic effects of ASD VI have revealed its potential for multitarget regulation of immune cells, but the depth and consistency of evidence need to be strengthened. The primary limitation of most of these studies is that they use acute inflammatory models induced by strong stimuli such as LPS, which is different from the long-term and low-grade inflammatory states of chronic inflammatory diseases like rheumatoid arthritis. In terms of mechanism, although multiple pathways like AMPK, Nrf2, and STAT3 are involved, most of the evidence is at the level of protein expression changes, and there is a lack of causal verifications using gene knockout or specific inhibitors to determine the core target pathways. In addition, research on the mechanisms of central analgesia are particularly weak, and the hypothesis that the GABA or adrenergic receptors are affected lacks direct functional evidence. Future research efforts should thus focus on validating the efficacy of ASD VI in chronic pain and inflammation models as well as using molecular pharmacological methods to elucidate the direct targets. At present, the analgesic and anti-inflammatory mechanisms of ASD VI need to be studied further. Details on the above works as well as their advantages and disadvantages are noted in Supplementary Table S7.

### 5.4 Neuroprotective effects

Alzheimer’s disease (AD) is a chronic neurodegenerative disorder that manifests as progressive cognitive deterioration, mnestic dysfunction, and cognitive-behavioral changes. Its main pathological markers are accumulation of A*β* protein in the form of amyloid plaques and hyperphosphorylation of tau protein manifesting as tau tangles, which eventually lead to progressive dementia. The clinical symptoms include learning, memory, and cognitive dysfunctions (Song et al., 2024). In the AD rat model induced by intracerebral injection of A*β* protein, Qian et al. (1999) discovered that ASD VI significantly inhibited the overexpression of A*β* in the neurons as well as the emission of cytokines and inflammatory factors from glial cells, thus inhibiting inflammatory responses and enhancing the learning capacity and memory functions in rats. Yu et al. (2012) further found that ASD VI could restore the learning capacity and memory function deficits of AD model rats; it could potentially inhibit as well as clear the deposition of B-AP in the dentate gyrus and CAI area of the hippocampal structure to protect nerve fibers. These behavioral and pathological improvements are encouraging, but the study did not explore the key issue of whether ASD VI could penetrate the blood–brain barrier and reach an effective concentration in the brain. Wang et al. (2018) demonstrated that ASD VI could reduce the level of corticosterone in the body by downregulating the hypothalamic–pituitary–adrenal (HPA) axis, thereby improving the memory deficit and anxiety state caused by A*β*_25-35_ in rats while offering neuroprotective effects to rats with cognitive dysfunction. Similarly, the studies lack assessments of the central drug exposure levels. Chen et al. (2013) discovered that ASD VI could downregulate the relative expressions of the *ECE2* and *α*2-giant globulin (*A2M*) genes to repair A*β*_25-35_-induced nerve cell damage. In addition, ASD VI could activate the PI3K/AKT signaling pathway, block the Notch signaling pathway, enhance the learning and cognitive memory abilities of sleep-deprived mice, and induce neuronal stem cells to differentiate into neurons to offer neuroprotective properties (Liu et al., 2020; Wang et al., 2023).

The neuroprotective effects of ASD VI in the AD model is of clinical concern, but its development as a neurotherapeutic drug involves severe challenges. The core problem lies in the gap between the model and disease complexity, where current studies mainly use acute injury models induced by A*β*_25-35_ hippocampal injection or scopolamine. These models only simulate a fragment of the pathology of AD and do not include tau protein lesions, neurofibrillary tangles, and persistent neuroinflammation. The most critical limitation is whether ASD VI can pass through the blood–brain barrier. As a highly polar saponin molecule, the central bioavailability of ASD VI is a fundamental issue that has not been studied. If there is not enough exposure to the brain, the *in vitro* neuroprotective effects are meaningless. In addition, existing studies focus more on short-term behavioral improvements but lack evaluations of the long-term therapeutic effects and pathological markers. Hence, future research must first confirm the blood–brain barrier penetration and evaluate the long-term efficacy in transgenic animal models such as 3xTg-AD. Details on the above works as well as their advantages and disadvantages are summarized in Supplementary Table S8.

### 5.5 Hepatoprotective effects

Non-alcoholic fatty liver disease (NAFLD) is a disorder that develops from simple fatty liver to non-alcoholic steatohepatitis (NASH) and further to cirrhosis. At present, the pathogenesis of NASH is complex and involves many factors, so it is not fully understood. The generally accepted idea is the classic “second shock” doctrine. Here, the “first shock” mainly refers to lipid deposition in the hepatocytes caused by insulin resistance and lipid metabolism disorder, leading to simple fatty liver (Zhao et al., 2020; Kazankov et al., 2019), while the “second shock” is pivotal to hepatocyte apoptosis (Zhang et al., 2023). Li (2014) confirmed that ASD VI could suppress the levels of the proapoptotic protein Bcl-2-associated X protein (Bax) by inhibiting phosphorylation of the JNK protein, facilitating the levels of the antiapoptotic protein Bcl-2, inhibiting the release of mitochondrial cytochrome C, and finally blocking the mitochondrial apoptosis pathway to inhibit the programmed death of hepatic cells. In a mouse model of NAFLD, Gong et al. (2014) found that ASD VI could improve mitochondrial respiratory damage, increase the levels of antiapoptotic protein Bcl-2, suppress the levels of Bax, and further inhibit rotenone-induced apoptosis of rat hepatocytes (BRL), suggesting that ASD VI could play a role in liver protection through the mitochondria. However, this study considered the classical apoptosis mechanism at the whole animal level but did not include a positive drug control, making it difficult to compare the efficacy horizontally. In the rotenone-induced rat liver cell injury model, Wei et al. (2023) found that ASD VI could restrain ERS by activating the AMPK signaling pathway, which had protective effects on alcoholic hepatic steatosis and liver injury in mice. In the mouse model of alcoholic liver injury, Gong et al. (2016) found that ASD VI activated autophagy flux by reducing microtubule-associated protein 1 LC3-II expression and p62 protein accumulation in the autophagosomes, significantly reducing liver fatty degeneration as well as liver cell apoptosis in ob/ob mice, indicating that ASD VI could protect the liver through autophagy regulation. These studies expand the application scenarios of ASD VI but fails to address the core transformation problem of its pharmacokinetic behaviors in the disease state.

The study of ASD VI in the NAFLD model reveals the possibility of its roles in regulating apoptosis, autophagy, and ERS. However, there are obvious faults in the existing evidence system. On the one hand, the mechanistic research is scattered in multiple pathways (JNK/Bcl-2, AMPK, and autophagic flux), but the interactions between each pathway and its primary and secondary roles in different pathological stages of NAFLD/NASH have not been clarified. On the other hand, the biggest obstacle to transformation is that the particular pharmacokinetic characteristics have not been considered in the context of liver disease. Liver disease itself can significantly change the metabolism and distribution of drugs, and it is unknown whether there is a difference in the disposal process of ASD VI between normal and steatotic livers. In addition, extant studies are mostly based on diet-induced early steatosis models, and there are insufficient data on the model efficacy for progression to the hepatitis and fibrosis stage. In the future, it is necessary to verify the efficacies in more advanced NASH models and carry out liver-specific pharmacokinetic studies in disease states. Details on the above works as well as their advantages and disadvantages are listed in Supplementary Table S9.

### 5.6 Cardioprotective effects

Over the past few years, advanced research on the pathophysiological mechanisms of myocardial ischemic injury have shown that it is closely related to cardiomyocyte apoptosis. Inhibition of cardiomyocyte apoptosis can effectively reduce myocardial ischemic injury (Xiang et al., 2024). PI3K plays an important role in the growth, differentiation, and apoptosis of cardiomyocytes. Activation of PI3K can activate its downstream substrate AKT, which directly phosphorylates various transcription factors like cyclic adenosine phosphate reaction binding protein (CREB). Modulation of these transcription factors can suppress the expressions of proapoptotic genes and upregulate the antiapoptotic genes, thereby facilitating cell survival (Feng et al., 2020). In the hypoxia/reoxygenation injury model of H9c2 cardiomyocytes, Li et al. (2010) found that ASD VI (10 and 20 μM) pretreatment could activate the hypoxia-induced AKT and CREB signaling pathways in the cardiomyocytes, significantly inhibit the expression of proapoptotic Bax and enhance the expression of antiapoptotic protein Bcl-2, and significantly inhibit the apoptosis of cardiomyocytes. This study reports a clear dose-effect relationship, but exploration of the timeliness of drug action and more downstream effect targets are insufficient. The study of H_2_O_2_-induced oxidative stress injury in H9c2 cells by Feng et al. (2020) and Liu et al. (2022) showed that ASD VI had a certain protective effect on HaCaT cells damaged by H_2_O_2_-induced oxidative stress; they confirmed that its mechanism was related to activating the ATF6 pathway, alleviating the H_2_O_2_-induced oxidative stress of H9c2 cells, and inhibiting apoptosis, all of which play myocardial protective roles. Animal experiments confirm that ASD VI can alleviate myocardial ischemic injury, improve cardiac role prognosis, and myocardial fibroplasia (Li et al., 2012).

The myocardial protective effects of ASD VI are mainly based on the hypoxia/reoxygenation and oxidative-stress-induced cell injury models, but the evidence for clinical transformation to cardiology is still unclear. The main limitations are that the research model is too simplified and that the current cell model cannot simulate the complex processes involved in myocardial ischemia-reperfusion injury *in vivo*, such as energy metabolism crisis, calcium overload, inflammatory cell infiltration, and microvascular dysfunction. Research on the action mechanisms focus on the common antiapoptotic endpoint, and there is still a lack of exploration of whether ASD VI has specific regulatory effects on myocardial cell metabolism, ion channels, or systolic functions. More importantly, pharmacological studies of the cardiovascular system rely heavily on clear pharmacokinetic and hemodynamic data, while the *in vivo* cardiac distribution of ASD VI as well as its potential effects on the blood pressure and heart rate are unknown. Future works must therefore be validated in animal models of myocardial infarction and systematically evaluated for safety and efficacy to cardiac functions and electrophysiology. Details on the above works as well as their advantages and disadvantages are given in Supplementary Table S10.

### 5.7 Other pharmacological effects

1. Enhancing blood flow and eliminating static blood via antithrombotic effect: In the model rats of Qi deficiency and blood stasis syndrome in TCM, Zhang Y. H. et al. (2020), Zhang and Ye (2021), and Deng and Liu (2020) found that ASD VI could significantly improve the survival rates and coordination abilities, significantly reduce the amounts of TNF-*α* and malondialdehyde in the body mass and cerebral tissues, as well as increase the activity of superoxide dismutase. These studies also found that ASD VI could reduce the dry and wet weights of thrombin in mice and rats, increase the concentration of 6-keto-F1*α* in plasma, decrease the concentration of TXB2, increase the ratio of 6-keto-F1*α*/TXB2, regulate the balance between 6-keto-F1*α* and TXB2 *in vivo*, and inhibit platelet aggregation. These evaluations based on the TCM syndrome model have certain characteristics, but the degree of model standardization and specificity of the evaluation index are still being discussed in academic circles.
2. Anticancer activity: Zhou et al. (2012) found that ASD VI could restrain cell growth and trigger apoptosis in U937 cells and HL-60 cells; they confirmed that its action mechanism was related to the decreased expression of the *Bcl-2* gene, upregulation of p53 protein, and promotion of NO content in the leukemia cells. However, this was only a preliminary study at the cellular level and is limited to two hematological tumor cell lines; its mechanistic exploration is superficial and lacks subsequent in-depth verification.
3. Treatment of tendinopathy: In animal models of tendon injury, ASD VI was found to restore the arrangement and distribution of fiber tissues, reduce the protein expression quantities of matrix metalloproteinase 1 and metalloproteinase restrain 1 in the tendon tissues, increase the protein expression quantities of TGF-*β*1 and PAI, confirm the mechanism and normalize the abnormal metabolism of extracellular matrix collagen, induce the proliferation of tendon cells, maintain the stability of tendon collagen, and promote healing of the damaged tendons (Wang and He, 2022).

The above pharmacological effects provide new directions for the study of new pharmacological effects of ASD VI and also application prospects for the clinical utilization of *D. asper*. However, the evidence is isolated and molecular mechanism descriptions remain vague, which need more independent confirmation studies. Details on the above works as well as their advantages and disadvantages are shown in Supplementary Table S11.

### 5.8 Results of bias risk assessment in the pharmacological studies

The bias risk assessment of the 11 original studies on the pharmacological activities of ASD VI (Supplementary Table S12) shows that the current evidence system had some systematic methodological weaknesses: the rigor of the experimental design was generally insufficient (approximately 90% of the studies had risks), which was mainly reflected in the vague sample size reports, lack of positive controls, and insufficient basis for dose selection basis; the models used in the studies were highly simplified, and approximately 70% of the studies relied on single cell lines or acute injury animal models; the pathological correlations of the models with complex human chronic diseases were doubtful and were rated as medium or high risk; more than 95% of the studies only reported the correlation changes of the signaling pathways, and there was a lack of key causality verification experiments, which resulted in the mechanistic depth being rated as moderate risk; there is a serious lack of self-criticism consciousness and most of the studies did not discuss their own limitations, which affects the objective interpretation of the evidence. These assessment results suggest that the existing evidence on the pharmacological effects of ASD VI is at a moderate risk of bias. Although the preliminary effects are clear, the robustness and clinical extrapolation value of the conclusions are restricted by the above methodological limitations (Supplementary Table S12).

The pharmacological effects (improving blood flow, antitumor effect, and tendinopathy treatment) reviewed in this section represent a new research dimension for ASD VI, but the relevant evidence is still preliminary in nature. The number of independent studies available for each effect is small. The relevant works are reported by only one or two teams for each case, lacking repeated verifications by different laboratories and confirmation of the reliability of the results. The research design is rough, such as the antithrombotic studies that have not been explored for selective effects on platelet aggregation induced by different agonists; anticancer research is limited to a few blood tumor cell lines, and the mechanisms are very superficial (only p53, Bcl-2); the research mechanism for the treatment of tendinopathy is vague (regulation of extracellular matrix metabolism). These directions lack follow-up research and thorough investigation of the mechanism. Therefore, it is too early to list these effects as the pharmacological effects of ASD VI, and a more appropriate positioning would be “potential tips for new activities.” Confirmation regarding new roles would require a large number of supporting follow-up, rigorous, and in-depth research works.

## 6 Conclusion and future prospects

To date, researchers have reported the extraction and recognition of 33 triterpenoid saponins, 21 iridoid glycosides, 16 phenolic acids, three alkaloids, six lignin metabolites, 53 essential oil components, polysaccharides, sucrose, vitamins, and inorganic elements like calcium, iron, magnesium, zinc, and copper from *D. asper*. Among these, ASD VI is the most prominent triterpenoid saponin in *D. asper* that holds great medicinal value; it can promote bone tissue growth, prevent RSA, relieve pain, as well as protect the nerves, liver, and cardiomyocytes. *D. asper* is often processed by sweating, stir-frying with wine, stir-frying with saltwater, and other methods to increase the content and dissolution rate of ASD VI while ensuring good pharmacological effects.

However, a critical analysis shows that there remain significant limitations to its broad usage and understanding. Most of the evidence supporting ASD VI are derived from single *in vitro* or animal models and that the mechanistic interpretations are mostly at the correlation level, lacking causality verifications. Crucially, there is almost no data on the pharmacokinetics, systemic toxicology, and clinical efficacy of ASD VI, which constitutes the fundamental bottleneck for its transformation into a modern drug.

Future research efforts should thus focus on the following priority directions: conducting systematic pharmacokinetic and safety evaluations; verifying the efficacy in a more clinically relevant disease model; elucidating the molecular mechanisms of component transformation and drug efficacy enhancement caused by processing using chemical biology; actively exploring delivery strategies to improve its bioavailability. In this review, we integrated and critically examined the complete evidence chain of chemical composition, processing modifications, and pharmacological mechanisms with the aim of providing a clear and reliable theoretical basis as well as roadmap for subsequent in-depth development and transformation applications of ASD VI. Although these efforts still rely on animal models to simulate the complex pathological functions, future research should prioritize the use of more advanced *in vitro* models or computational simulation methods to reduce the use of animals in research.
