# Supplementary material for: Research progress on chemical metabolites, processing technologies, and pharmacological activities of asperosaponin VI: a systematic review and critical evaluation
Source: Front Pharmacol. 2026 Apr 10;17:1725604. doi: 10.3389/fphar.2026.1725604 (PMC13106191; doi:10.3389/fphar.2026.1725604)
Supplement: Supplementary file 1 [file DataSheet1.pdf]

Directory list

TYPE	Serial Number	Content
Figure	1	Chemical structure of triterpenoid saponins form *Dipsacus asper*
	2	Chemical Structure of iridoid glycosides in *Dipsacus asper*
	3	Chemical Structure of phenolic acids in *Dipsacus asper*
	4	Chemical structure of alkaloids in *Dipsacus asper*
	5	Chemical structure of lignins in *Dipsacus asper*
	6	Content of ASD VI in *Dipsacus asper*
	7	*Dipsacus asper* processed products (left is Sudation, and rest are stir-frying with wine.)
Table	1	Triterpenoid saponins in *Dipsacus asper*
	2	Iridoid glycosides in *Dipsacus asper*
	3	Phenolic acids in *Dipsacus asper*
	4	oklueoil plkalolds in *Dipsacus asper*
	5	ASD VI research data summary Table in the osteoporosis field
	6	Summary of pharmacological studies on ASD VI for preventing recurrent spontaneous abortion (RSA)

7	Summary of analgesic and anti-inflammatory studies on ASD VI
8	ASD VI research data summary Table in the AD field
9	Summary of hepatoprotective studies on ASD VI
10	Summary of cardioprotective studies on ASD VI
11	Summary of other pharmacological studies on ASD VI
12	ASD VI multi-field research (bone/neurology/liver/cardiovascular) risk assessment Table

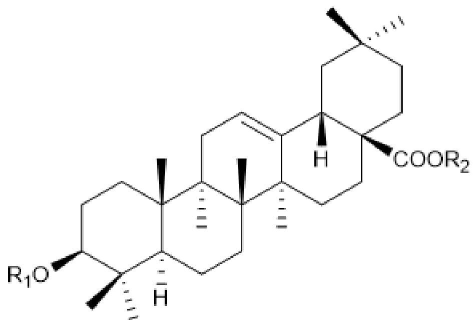

Figure 1 Chemical structure of triterpenoid saponins form *Dipsacus asper*

Table 1 Triterpenoid saponins in *Dipsacus asper*

Number	Name	The side chain (R1-R3 in **Error! Reference source not found.**)			Source	Site	Document
		R1	R2	R3			
1	Hederagenin	H	OH	H	*Dipsacus asper*	Roots	Wei and Liang, (1987)
2	Dipsacus saponin IV	4-*O*-acetyl-ara	OH	Glc(6→1)glc	*Dipsacus asper*	Roots	Zhang and Xue, (1991a)
3	Dipsacus saponin V	Ara	H	Glc(6→1)glc	*Dipsacus asper*	Roots	Zhang and Xue, (1991a)

Number	Name	The side chain (R1-R3 in **Error! Reference source not found.**)			Source	Site	Document
		R1	R2	R3			
4	Dipsacus saponin VI	Ara	OH	Glc(6→1)glc	*Dipsacus asper*	Roots	Zhang and Xue, (1991a)
5	Macranthoidin A	Glc(1→3)rha(1→2)ara	OH	Glc(6→1)glc	*Dipsacus asper*	Roots	Zhang and Xue, (1991 b)
6	Dipsacus saponin VIII	Rha(1→3)glc(1→3)rha(1→2)ara	OH	Glc(6→1)glc	*Dipsacus asper*	Roots	Zhang and Xue, (1991 b)
7	Dipsacus saponin IX	Xyl(1→4)glc(1→4)[rha(1→3)]glc(1→3)rha(1 →2)ara	OH	H	*Dipsacus asper*	Roots	Zhang and Xue, (1992)
8	Dipsacus saponin X	Xyl(1→4)glc(1→4)[rha(1→3)]glc(1→3)rha(1 →2)ara	OH	Glc(6→1)glc	*Dipsacus asper*	Roots	Zhang and Xue, (1992)
9	Dipsacus saponin XI	Glc(1→4)[rha(1→3)]glc(1→3)rha(1→2)ara	OH	H	*Dipsacus asper*	Roots	Zhang and Xue, (1993)
10	Dipacus saponin XII	Glc(1→4)[rha(1→3)]glc(1→3)rha(1→2)ara	OH	Glc(6→1)glc	*Dipsacus asper*	Roots	Zhang and Xue, (1993)

Number	Name	The side chain (R1-R3 in **Error! Reference source not found.**)			Source	Site	Document
		R1	R2	R3			
11	Dipsacus saponin XIII	Xyl(1→4)glc(1→4)[rha(1→3)]glc(1→3)rha(1→2)ara	H	Glc(6→1)glc	*Dipsacus asper*	Roots	Zhang and Xue, (1993)
12	Dipsacus saponin A	H	OH	Glc(6→1)glc	*Dipsacus asper*	Roots	Yang *et al*., (1993)
13	Cauloside A	Ara	OH	H	*Dipsacus asper*	Roots	Yang *et al*., (1993)
14	HN saponin F	Ara	OH	Glc	*Dipsacus asper*	Roots	Yang *et al*., (1993)
15	3-*O*-[*β*-*D*-xylopyranosyl(1→4)-*β*-*D*-glucopyranosy1(1→4)][*α*-*L*-rhamnopyranosyl(1→3)]-*β*-*D*-glucopyranosyl(1→3)-*α*-*L*-rhamnopyranosyl(1→2)-*α*-*L*-arabinopyranosyl-hederagenin-28-*O*-*β*-*D*-glucopyrano side	Xyl(1→4)glc(1→4)[rha(1→3)]glc(1→3)rha(1→2)ara	OH	Glc	*Dipsacus asper*	Roots	Yang *et al*., (1993)
16	Dipsacus saponin B	Glc(1→4)[rha(1→6)]glc(1→3)rha(1→2)ara	OH	H	*Dipsacus asper*	Roots	Jung *et al*.,

Number	Name	The side chain (R1-R3 in **Error! Reference source not found.**)			Source	Site	Document
		R1	R2	R3			
							(1993)
17	Dipsacus saponin C	Xyl(1→4)glc(1→4)glc(1→3)[rha(1→3)]rha(1→2)ara	OH	H	*Dipsacus asper*	Roots	Jung *et al*., (1993)
18	Macranthoside B	Glc(1→4)glc(1→3)rha(1→2)ara	OH	H	*Dipsacus asper*	Roots	Jung *et al*., (1993)
19	3-*O*-*β*-*D*-xylopyranosyl(1→4)-*β*-*D*-glucopyranosyl(1→4)-*β*-*D*-glucopyranosy(1→3)-*α*-*L*-rhamnopyranosyl(1→2)-*α*-*L*-arabinopyranosyl-hederagenin	Xyl(1→4)glc(1→4)glc(1→3)rha(1→2)ara	OH	H	*Dipsacus asper*	Roots	Jung *et al*., (1993)
20	Dipsacus saponin F	Xyl(1→4)glc(1→4)[rha(1→3)]gal(1→3)rha(1→2)ara	OH	H	*Dipsacus asper*	Roots	Wei *et al*., (1994)
21	Dipsacus saponin H_1	Xyl(1→4)glc(1→4)[rha(1→3)]gal(1→3)rha(1→2)ara	OH	Glc(6→1)glc	*Dipsacus asper*	Roots	Wei *et al*., (1994)
22	3-*O*-*β*-*D*-glucopyranosyl(1→3)-*α*-Lrhamnopyranosyl(1→2)-*β*-*L*	Glc(1→3)rha(1→2)ara*	OH	Glc(6→1)glc	*Dipsacus asper*	Roots	Oh *et al*., (1999b)

Number	Name	The side chain (R1-R3 in **Error! Reference source not found.**)			Source	Site	Document
		R1	R2	R3			
	-arabinopyranosyl-hederagenin-28-*O*-*β*-*D*-glucopyranosyl(1→6)-*β*-*D*-glucopyranoside						
23	3-*O*-*β*-*D*-glucopyranosyl(1→3)[*α*-*L*-rhamnopyranosy1(1→2)]*α*-*L*-arabinopyranosyl-hederagenin-28-*O*-*β*-*D*-glucopyranosyl(1→6)-*β*-*D*-glucopyranoside	Glc(1→3)[rha(1→2)]ara	OH	Glc(6→1)glc	*Dipsacus asper*	Roots	Oh *et al.*, (1999b)
24	3-*O*-*α*-*L*-rhamnopyranosy(1→3)-*β*-*D*-glucopyranosyl(1→3)-*α*-*L*-rhamnopyranosyl(1→2)-*α*-*L*-arabinopyranosyl-hederagenin	Rha(1→3)glc(1→3)rha(1→2)ara	OH	H	*Dipsacus asper*	Roots	Miu *et al.*, (1999)
25	3-*O*-*β*-*D*-glucopyranosyl(1→4)[*α*-*L*-rhamnopyranosy1(1→3)]-*β*-*D*-glucopyranosyl(1→3)-*α*-*L*-rhamnopyranosyl(1→2)-*β*-*L*-ara	Glc(1→4)[rha(1→3)]glc(1→3)rha(1→2)ara*	OH	Glc(6→1)glc	*Dipsacus asper*	Roots	Miu *et al.*, (1999)

Number	Name	The side chain (R1-R3 in **Error! Reference source not found.**)			Source	Site	Document
		R1	R2	R3			
	binopyranosyl-hederagenin-28-*O*-*β*-*D*-glucopyranosyl(1→6)-*β*-*D*-glucopyranoside						
26	Kalopanaxsaponin A	Rha(1→2)ara.	OH	H	*Dipsacus asper*	Roots	Hung *et al*., (2005)
27	3-*O*-*β*-*D*-xylopyranosyl(1→3)-*α*-*L*-rhamnopyranosyl(1→2)-*α*-*L*-arabinopyranosyl-hederagenin	Xyl(1→3)rha(1→2)ara	OH	H	*Dipsacus asper*	Roots	Hung *et al*., (2005)
28	3-*O*-*β*-*D*-glucopyranosyl(1→3)-*α*-*L*-rhamnopyranosyl(1→2)-*α*-*L*-arabinopyranosyl-hederagenin	Glc(1→3)rha(1→2)ara	OH	H	*Dipsacus asper*	Roots	Hung *et al*., (2005)
29	Oleanolic acid	H	H	H	*Dipsacus asper*	Roots	Tian *et al*., (2007)
30	Dipsacus saponin J	Glc(1→4)[rha(1→3)]glc(1→3)rha(1→2)ara	OH	Glc	*Dipsacus asper*	Roots	Liu *et al*., (2011)

Number	Name	The side chain (R1-R3 in **Error! Reference source not found.**)			Source	Site	Document
		R1	R2	R3			
31	Dipsacus saponin K	Ara	OH	Glc(6→1)glc(6→1) glc	*Dipsacus asper*	Roots	Liu *et al.*, (2011)
32	Daucosterol	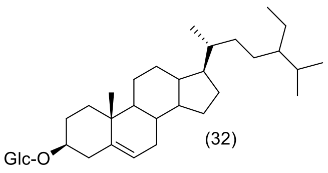	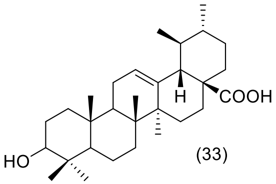		*Dipsacus asper*	Roots and aboveground parts	Zhang and Xue, (1991a)
33	Ursolic acid				*Dipsacus asper*		Wang *et al.*, (2006)

Ara*=*β*-*L*-arabinopyranosyl

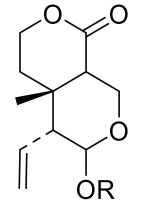

(34) R=Glc
(44) R=Glc(3→1)glc
(45) R=Glc(6→1)api

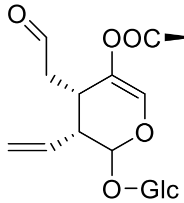
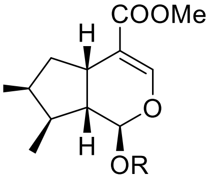

(36) R=Glc
(40) R=H

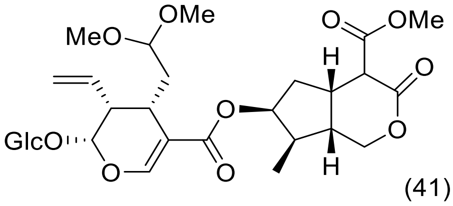

(41)

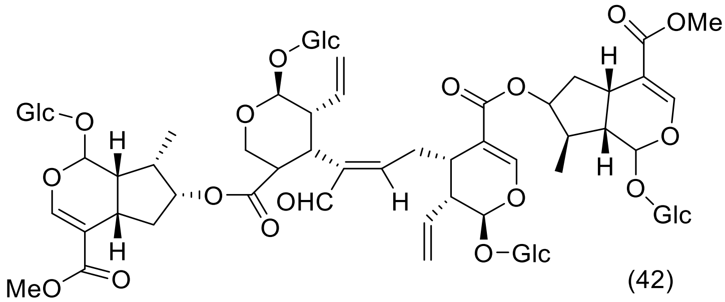

(42)

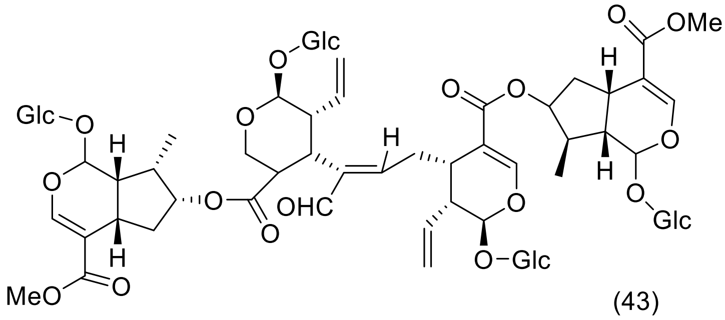

(43)

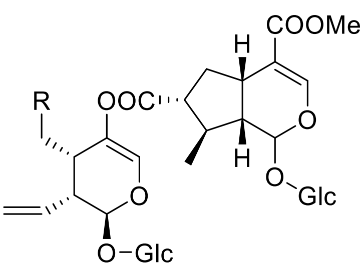

R= 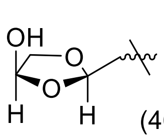 (46) 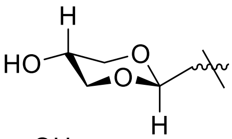 (48)

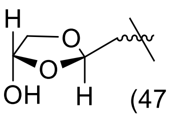 (47) 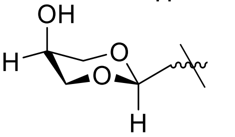 (49)

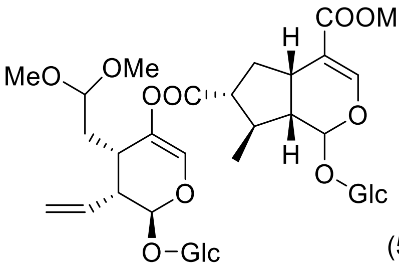

(50)

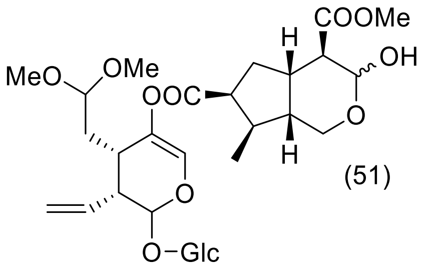

(51)

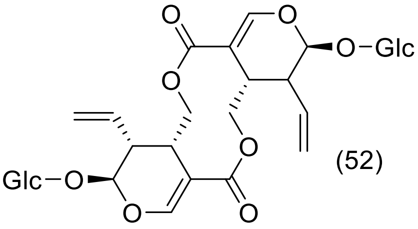

(52)

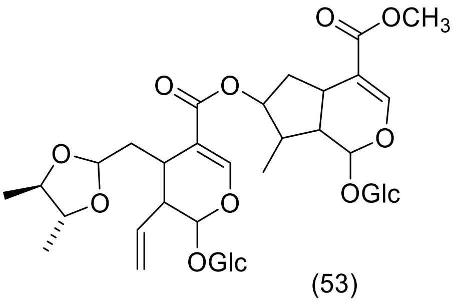

(53)

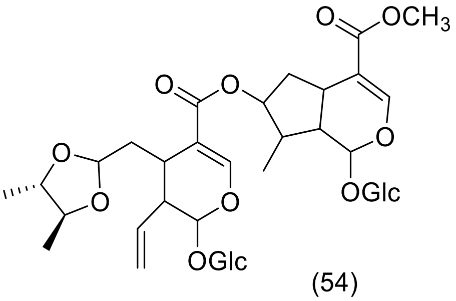

(54)

Figure 2 Chemical structure of iridoid glycosides in *Dipsacus asper*

Table 2 Iridoid glycosides in *Dipsacus asper*

Number	Name	Source	Site	Document
34	Sweroside	*Dipsacus asper*	Roots, aerial parts and seeds	Kauno *et al*., (1990)
35	Loganin	*Dipsacus asper*	Roots, aerial parts and seeds	Kauno *et al*., (1990)
36	Cantleyoside	*Dipsacus asper*	Roots, aerial parts and seeds	(Kauno *et al*., 1990)

Number	Name	Source	Site	Document
37	6'-*O*-*β*-*D*-Glucopyranosylloganin	*Dipsacus asper*	Roots	Tomita and Mouri, (1996)
38	Loganic acid	*Dipsacus asper*	Roots	Tomita and Mouri, (1996)
39	6'-*O*-*β*-*D*-Glucopyranosylloganic acid	*Dipsacus asper*	Roots	Tomita and Mouri, (1996)
40	Sylvestroside III	*Dipsacus asper*	Roots	Wei and Lou, (1996)
41	Sylvestroside IV dimethylacetal	*Dipsacus asper*	Aboveground parts	Tomassini *et al*., (2004)
42	Dipsanoside A	*Dipsacus asper*	Roots	Tian *et al*., (2006)
43	Dipsanoside B	*Dipsacus asper*	Roots	Tian *et al*., (2006)
44	Dipsanoside H	*Dipsacus asper*	Roots	Tian *et al*., (2007)
45	6'-*O*-*β*-*D*-Apiofuranosyl sweroside	*Dipsacus asper*	Roots	Tian *et al*., (2007)
46	Dipsanoside C	*Dipsacus asper*	Roots	Tian *et al*., (2007)
47	Dipsanoside D	*Dipsacus asper*	Roots	Tian *et al*., (2007)
48	Dipsanoside E	*Dipsacus asper*	Roots	Tian *et al*., (2007)
49	Dipsanoside F	*Dipsacus asper*	Roots	Tian *et al*., (2007)
50	Triplostoside A	*Dipsacus asper*	Roots	Tian *et al*., (2007)
51	Dipsanoside G	*Dipsacus asper*	Roots	Tian *et al*., (2007)
52	Lisianthioside	*Dipsacus asper*	Roots	Tian *et al*., (2007)
53	Dipsanoside M	*Dipsacus asper*	Roots	Sun *et al*., (2015)
54	Dipsanoside N	*Dipsacus asper*	Roots	Sun *et al*., (2015)

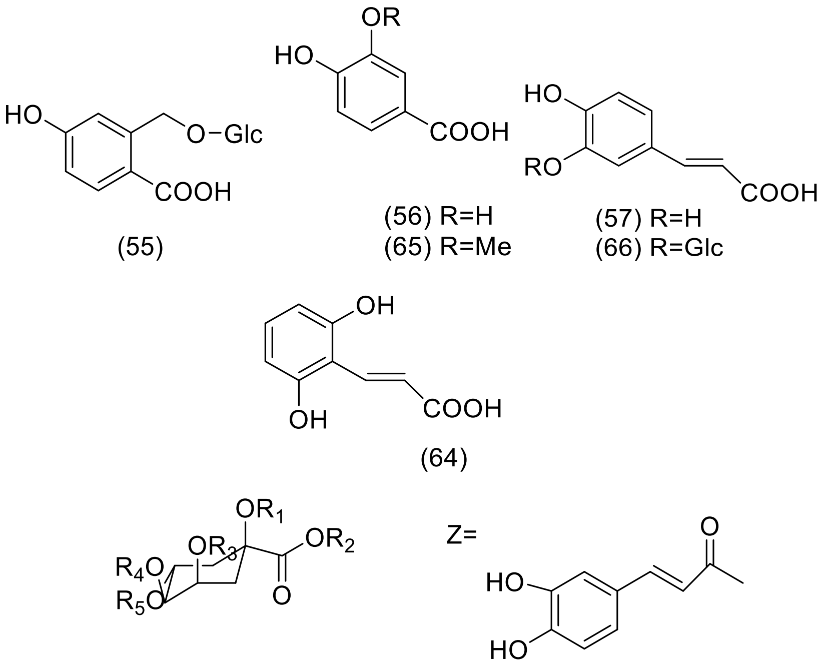

(58) R_1=H R_2=H R_3=Z R_4=Z R_5=H
(59) R_1=H R_2=Me R_3=Z R_4=Z R_5=H
(60) R_1=H R_2=H R_3=Z R_4=H R_5=Z
(61) R_1=H R_2=Me R_3=Z R_4=H R_5=Z
(62) R_1=H R_2=H R_3=H R_4=Z R_5=Z
(63) R_1=H R_2=Me R_3=H R_4=Z R_5=Z
(67) R_1=Z R_2=H R_3=H R_4=H R_5=H
(68) R_1=H R_2=H R_3=H R_4=H R_5=Z
(69) R_1=H R_2=H R_3=H R_4=Z R_5=H
(70) R_1=H R_2=H R_3=H R_4=H R_5=Z

Figure 3 Chemical structure of phenolic acids in *Dipsacus asper*

Table 3 Phenolic acids in *Dipsacus asper*

Number	Name	Source	Site	Document
55	Dipsaicin	*Dipsacus asper*	Roots	Abdallah, (1991)
56	Protocatechuic acid	*Dipsacus asper*	Roots	(Kim *et al*., 1999)
57	Caffeic acid	*Dipsacus asper*	Roots	(Kim *et al*., 1999)
58	3,4-di-*O*-Caffeoylquinic acid	*Dipsacus asper*	Roots	(Hung *et al*., 2005)
59	Methyl3,4-di-*O*-caffeoylquinate	*Dipsacus asper*	Roots	(Hung *et al*., 2005)
60	3.5-di-*O*-Caffeoyquinic acid	*Dipsacus asper*	Roots	(Hung *et al*., 2005)
61	Methyl 3,5-di-*O*-caffeoylquinate	*Dipsacus asper*	Roots	(Hung *et al*., 2005)
62	4.5-di-*O*-Caffeoylquinic acid	Dipsacus asper	Roots	(Hung *et al*., 2005)

Number	Name	Source	Site	Document
63	Methyl4, 5-di-*O*-caffeoylquinate	*Dipsacus asper*	Roots	(Hung *et al*., 2005)
64	2,6-Dihydroxycinnamic acid	*Dipsacus asper*	Roots	(Tian *et al*., 2007)
65	Vanillicacid	*Dipsacus asper*	Roots	(Tian *et al*., 2007)
66	2'-*O*-Caffeoyl-*D*-glucopyranoside ester	*Dipsacus asper*	Roots	(Tian *et al*., 2007)
67	Caffeoylquinic acid	*Dipsacus asper*	Roots	(Tian *et al*., 2007)
68	Chlorogenic acid	*Dipsacus asper*	Roots	(Cao *et al*., 2010)
69	5-caffeoylquinic acid	*Dipsacus asper*	Roots	(Wang *et al*., 2014)
70	4-caffeoylquinic acid	*Dipsacus asper*	Roots	(Tao *et al*., 2019)

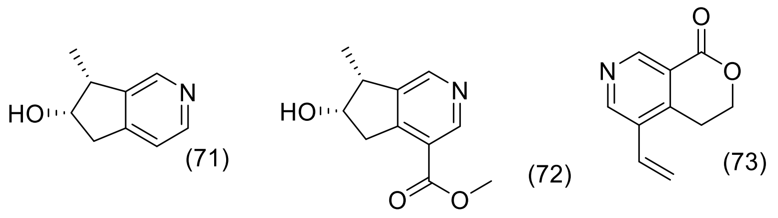

Figure 4 Chemical structure of alkaloids in *Dipsacus asper*

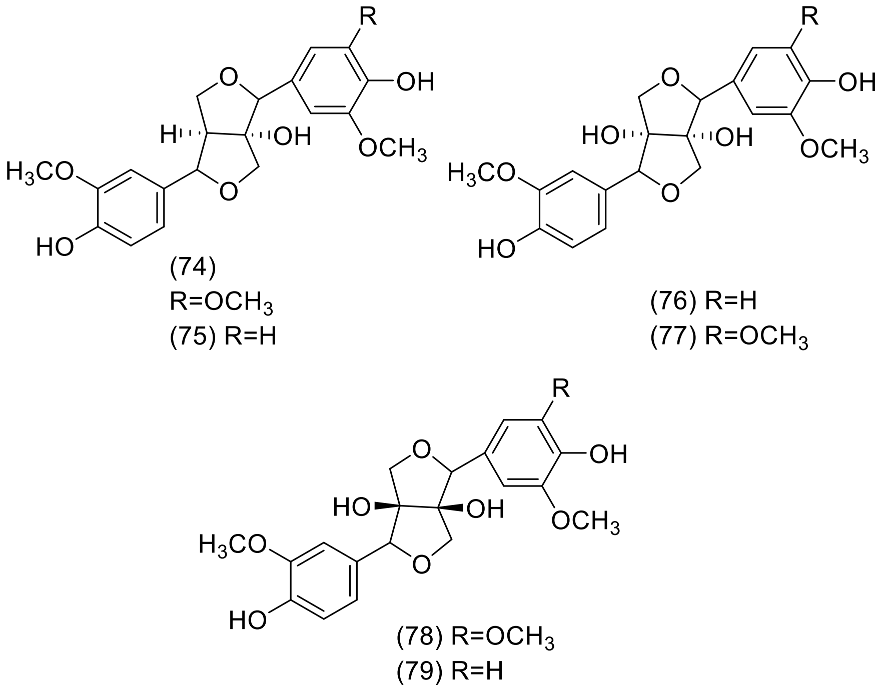

Figure 5 Chemical structure of lignins in *Dipsacus asper*

Table 4 Voklueoil plkalolds in *Dipsacus asper*

Name	Molecular formula	molecular weight	Name	Molecular formula	molecular weight
Hexanal	$C_6H_{12}O$	100	n-Heptadecane	$C_{17}H_{36}$	240
2,4-Dimethylhexane	C_8H_{18}	114	n-Eicosane	$C_{20}H_{42}$	282
3-Perhydroxyhexane	$C_6H_{14}O_2$	118	2,6,10-Trimethyldodecane	$C_{15}H_{32}$	212
Isodecane	$C_{10}H_{22}$	142	10-Methylnonadecane	$C_{20}H_{42}$	282
3-Methylene-1,7-octadiene	C_9H_{14}	122	Di-(2-ethylexyl)phthalate	$C_{24}H_{38}O_4$	390
Decane	$C_{10}H_{22}$	142	11-Decyltetracosane	$C_{34}H_{70}$	478
Nonanal	$C_9H_{18}O$	142	2,6,10,15-Tetramethylheptadecane	$C_{21}H_{44}$	296
2,6-Dimethylheptadecane	$C_{19}H_{40}$	268	Farnesol	$C_{15}H_{26}O$	222
Dodecane	$C_{12}H_{26}$	170	n-Tetracontane	$C_{44}H_{90}$	618
Tridecane	$C_{13}H_{28}$	184	n-Heneicosane	$C_{21}H_{44}$	296
2,6,10,14-Tetramethylheptadecane	$C_{21}H_{44}$	296	Tritetracontane	$C_{43}H_{88}$	604
6-Methyltridecane	$C_{14}H_{30}$	198	Palmitic acid-3-bromo-2-propyne ester	$C_{19}H_{33}BrO_2$	372
2,6,10-Trimethylpentadecane	$C_{18}H_{38}$	254	Cholesteryl myristate ester	$C_{41}H_{72}O_2$	596

Pentadecanoic acid	$C_{15}H_{30}O_2$	242	1-Chloroheptacosane	$C_{27}H_{55}Cl$	414
Ethyl eicosanoate	$C_{22}H_{44}O_2$	340	24-Methyl-5-cholesten-3-ol	$C_{28}H_{48}O$	400
3,5-Dimethyldodecane	$C_{14}H_{30}$	198	Stigmasterol	$C_{29}H_{48}O$	412
2,7,10-Trimethyldodecane	$C_{15}H_{32}$	212	1-Iodotetracontane	$C_{48}H_{97}$	800
11,14-Eicosadienoic acid Methyl ester	$C_{21}H_{38}O_2$	322	γ-Sitosterol	$C_{29}H_{50}O$	414
Z-7-Tetradecenal	$C_{14}H_{26}O$	210	Aurantioidein bromide	$C_{15}H_{25}Br$	284
12-Methyl-(E,E)-2,13-octadecadien-1-ol	$C_{19}H_{36}O$	280	1-chloroeicosane	$C_{20}H_{41}Cl$	316
Pentadecane	$C_{15}H_{32}$	212	Lanosterol	$C_{30}H_{50}O$	426
10-Methyleicosane	$C_{21}H_{44}$	296	4-Cholestene-3β-ol	$C_{32}H_{52}O_2$	468
6-Methyl-2-phenylquinoline	$C_{16}H_{13}N$	219	Lupeol	$C_{30}H_{50}O$	426
3,9-Dimethylundecane	$C_{13}H_{28}$	184	Urs-12-ene-28-al	$C_{30}H_{48}O$	424
2-Methyl-octadecane	$C_{19}H_{40}$	268	4α,14-dimethyl-9β,19-cyclo-5α-ergosta-24(28)-en-3 β-olacetate	$C_{32}H_{52}O_2$	468
Cetane	$C_{16}H_{34}$	226			

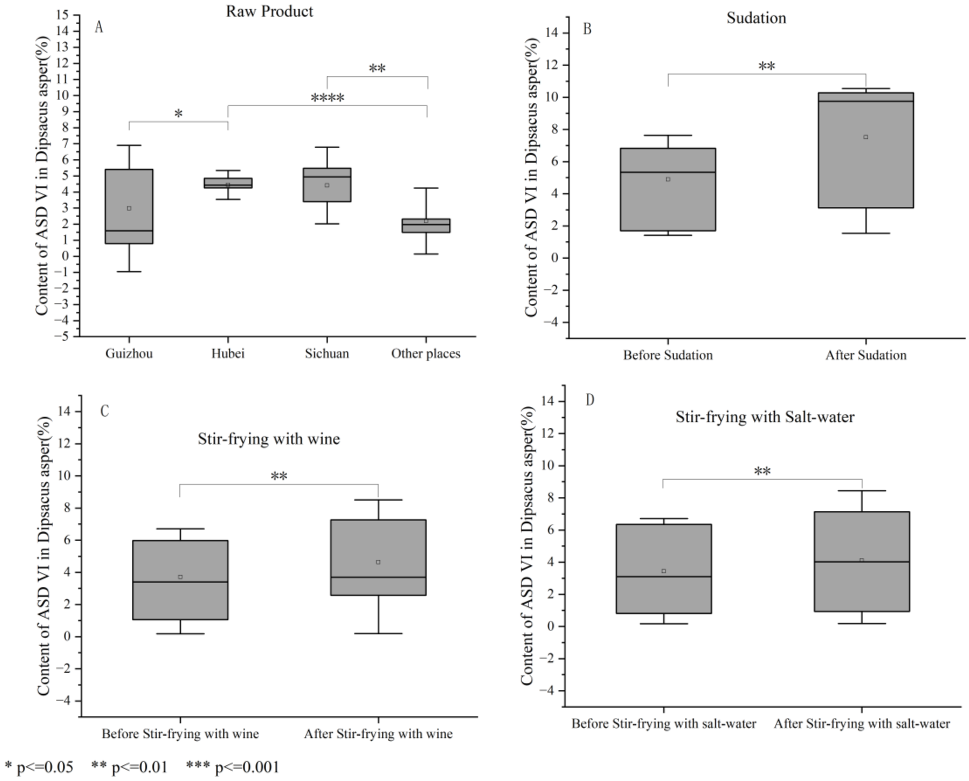

Figure 6 Content of ASD VI in *Dipsacus asper*

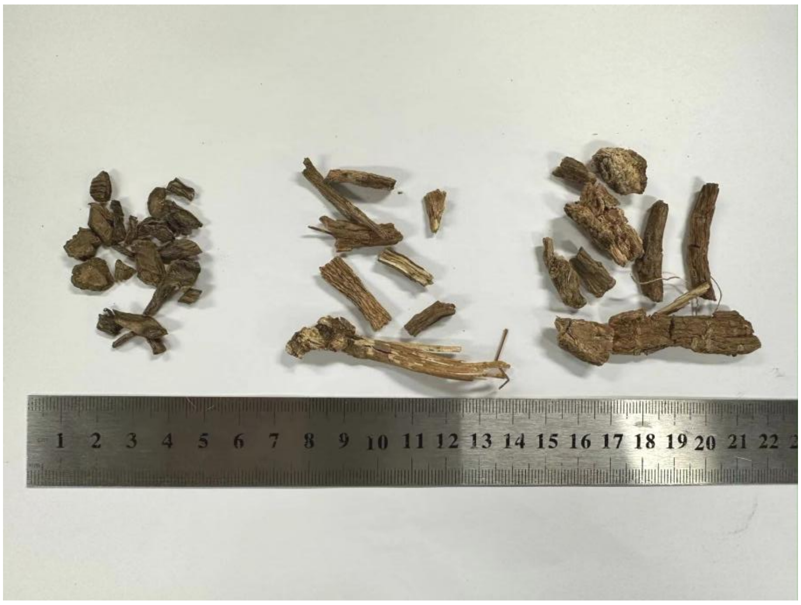

Figure 7 *Dipsacus asper* processed products (left is sudation, and rest are stir-frying with wine.)

Table 5 ASD VI research data summary Table in the osteoporosis field

Reference	Study Model	ASD VI Dose/Concentration	Key Findings	Critical Appraisal by This Review
Zhang et al. (2020)	Mouse bone marrow mesenchymal stem cells (BMSCs) under glucocorticoid	0.1, 1, 10 μM	Promotes osteogenic differentiation, Activates BMP/Smad pathway	**Strength**: A clear background of glucocorticoid injury was set, with pathological relevance.
				**Limitation**: Late-stage osteogenic differentiation markers (such as mineralized nodules) were not detected, and the mechanism was not using pathway inhibitor validation.
Niu et al. (2015)	Rat osteoblastic cell line (ROS 17/2.8 cells)	1, 10, 50 μg/mL	Promotes proliferation and ALP activity, Activates PI3K/AKT pathway	**Strength**: Cell proliferation and early differentiation markers were detected.
				**Limitation**: The concentration unit was μg/mL rather than molar concentration, which was not conducive to cross-study comparison. compound purity.
Liu et al (2019)	Collagen-induced arthritis (CIA) Mouse model in vivo	20, 40 mg/kg/day, oral gavage	Reduces bone destruction, Inhibits osteoclast formation	**Strength**: Validate the efficacy in the complex disease model of autoimmune inflammation.

			Wnt/β-catenin pathway	**Limitation**: Animal randomization method and blinding assessment details were not reported. Experimental rigor could be enhanced.
Ma et al. (2022) (Review)	N/A	N/A	Summarizes that ASD VI promotes bone remodeling through multiple pathways	**Strength**: Systematic integration.<br>**Limitation**: As a secondary literature, the reliability of its conclusions depends on the quality of the primary research cited.

Table 6 Summary of pharmacological studies on ASD VI for preventing recurrent spontaneous abortion (RSA)

Reference	Study model	ASD VI Dose/concentration	Key findings	Critical appraisal by this review
Gao et al. (2016)	*In vitro*: Primary decidual cells, HeLa cells	50, 100 and 200 μM	Activates progesterone receptor (PR), upregulates PR	Strength: Explored the molecular mechanism of the traditional “tocolysis” effect.

			expression, and initiates the Notch signaling pathway to promote decidualization.	Limitation: Uses HeLa (cervical cancer) cell line, whose relevance in pregnancy biology is limited. Mechanism remains at the correlative level.
Gao et al. (2016)	*In vitro*: Primary decidual cells, HeLa cells	1, 10, 100 μM	Upregulates PSG1 and PR expression, activates the AKT pathway, upregulates Bcl-2 and downregulates Bax.	Strength: Provides a multi-target mechanism for anti-abortion effect.
				Limitation: Same model limitation as above. Lacking direct molecular target verification.
Du Xin (2018)	*In vivo*: Mifepristone-induced	200 mM	Exerts anti-abortive effect (inferred to be related to PR	Strength: Validates efficacy in a hormone-intervention animal model.

	abortion mouse model		activation based on cellular studies).	Limitation: Key experimental details (animal number, treatment duration) are not reported in the review, limiting the assessment of rigor.

Table7 Summary of analgesic and anti-inflammatory studies on ASD VI

Reference	Study Model	ASD VI Dose/concentration	Key Findings	Critical appraisal by this review
Xuan et al. (2024)	*In vivo*: Allergic airway inflammation mouse model	50, 100 and 200 μM	Increases p-AMPK expression in lung tissue, exerting anti-inflammatory effect by inducing AMPK activation.	Strength: Explores a new therapeutic scenario (airway inflammation).
				Limitation: Whether AMPK activation is direct or indirect

				remains unclear. Lack of positive control.
Luo Jinfang et al. (2023)	*In vitro*: LPS-stimulated macrophages	5, 10, 20 μM	Inhibits M1-type polarization and promotes M2-type polarization, regulating M1/M2 balance for anti-inflammatory immunomodulation.	Strength: Identifies immunomodulation via macrophage phenotype switching as a key mechanism.<br>Limitation: Acute, strong inflammatory model differs from the chronic inflammation pathology.
Gong et al. (2019)	*In vitro*: Macrophage model	30, 60, 120mg/kg/d	Exerts anti-inflammatory activity by inhibiting the activation of the IL-6/STAT3 pathway	Strength: Reveals involvement of epigenetic regulation (DNMT3b).<br>Limitation: Limited to *in vitro* evidence. Causal relationship

			and downregulating DNMT3b expression.	needs verification with inhibitors/knockout.
Suh et al. (1996)	*In vivo*: Analgesia model (type unspecified)	3.75~30 μg	The analgesic effect may be linked to the modulation of GABA receptors and adrenergic receptors.	Limitation: This is a very early and preliminary study. The model and specific mechanisms are vaguely described, serving only as a potential clue.

Table 8 ASD VI research data summary Table in the AD field

Reference	Study Model	ASD VI Dose/Concentration	Key Findings	Evidence Quality Assessment
Qian et al., 1999	Aβ-induced Alzheimer's disease (AD) model rats	50, 100 mg/kg, Intraperitoneal Injection (*i.p.*)	Inhibits excessive Aβ expression in hippocampal neurons, Improves learning and memory abilities.	**Strength**: Early exploratory study that established the association between ASD VI and Aβ, a key pathological protein in AD.<br>**Limitation**: Acute intracerebral Aβ injection model was used,

				which was inconsistent with AD chronic course. Blinding assessment without behavioral tests ; the mechanism of action is unknown.
Yu et al., 2012	$A\beta_{25\text{-}35}$-induced Alzheimer's disease (AD) Model Rats	25, 50 mg/kg, oral gavage (*p.o.*)	Improves learning and memory deficits, Reduces Aβ deposition in hippocampal dentate gyrus and CA1 region.	**Strength**: Adopted oral administration with higher clinical relevance; focused on pathological improvement in specific brain regions.
				**Limitation**: Drug plasma and brain concentrations were not detected, and the relationship between efficacy and in vivo exposure was unknown; mechanism research is relatively superficial.
Zhou et al., 2009	$A\beta_{25\text{-}35}$-injured PC12 cells	0.1, 1, 10 μM	Increases cell viability, Reduces apoptosis rate, Protects neural synapses.	**Strength**: Clearly demonstrated the direct neuroprotective effect of ASD VI at the cellular level and provided a clear concentration gradient.

				**Limitation**: PC12 cells are pheochromocytoma cell line, and their neuronal characteristics are limited. The related signaling pathway mechanism was not explored.
Wang et al., 2018	Aβ_{25-35}-induced Cognitive Impairment Rats	20, 40 mg/kg, oral gavage (*p.o.*)	Downregulates HPA axis activity, Reduces corticosterone levels, Improves memory deficits and anxiety-like behaviors.	**Strength**: introduces a novel mechanism perspective of neuroendocrine (HPA axis) regulation, which broadens the understanding of mechanism of action.
				**Limitation**: HPA axis regulation is a systemic effect, and its direct protective effect on the central nervous system accounts for an unclear proportion; there is also a lack of pharmacokinetic data.
Liu et al., 2020	Drug-containing serum on neural stem cells	Serum from ASD VI-treated rats	Promotes neural stem cell proliferation, activates PI3K/AKT pathway and induces	**Strength**: Using the ‘drug-containing serum’ method, closer to the real form of action after in vivo metabolism; focus on neuroregenerative potential.

			differentiation into neurons.	**Limitation**: specific active ingredients in serum are unknown; the evidence that the effect is entirely attributable to ASD VI is not direct enough.
Wang et al., 2023	Sleep-deprived mouse model	20, 40 mg/kg, oral gavage (*p.o.*)	Improves neurogenesis and cognitive function, the mechanism is related to blocking Notch signaling pathway.	**Strength**: Explored sleep deprivation as a new injury model with practical significance; mechanism research points to the specific Notch pathway.
				**Limitation**: The cognitive impairment caused by sleep deprivation model is different from the pathological nature of neurodegenerative diseases such as AD, and extrapolation needs to be cautious.

Table 9. Summary of hepatoprotective studies on ASD VI

Reference	Study model	ASD VI Dose/concentration	Key Findings	Critical appraisal by this review
Li Guangrun (2014)	*In vivo*: Non-alcoholic fatty liver disease (NAFLD) mouse model	30, 60, 120 mg/kg/d	Inhibits JNK phosphorylation, modulates Bcl-2/Bax, and blocks the mitochondrial apoptosis pathway.	Strength: Explains the anti-apoptotic mechanism in an *in vivo* disease model.
				Limitation: Lack of positive control makes efficacy comparison difficult. Pharmacokinetics in steatotic liver unknown.
Gong et al. (2014)	*In vivo*: NAFLD mouse model	1, 10, 100 μM	Improves mitochondrial respiratory damage and regulates Bcl-2/Bax.	Strength: Supports the key role of mitochondria in the hepatoprotective effect.
				Limitation: Similar to the above, experimental details are insufficiently reported.

Wei et al. (2023)	*In vivo*: Alcoholic liver injury mouse model	50~100 μM	Inhibits endoplasmic reticulum stress (ERS) by activating the AMPK signaling pathway.	Strength: Expands the protective mechanism to the ERS pathway in a different etiology model.
				Limitation: Focuses on early steatosis; efficacy in advanced NASH/fibrosis models is unknown.
Gong et al. (2016)	*In vivo*: Alcoholic liver injury in ob/ob mice	10, 100 μM	Activates autophagy flux, reducing hepatic steatosis and apoptosis.	Strength: Reveals the regulation of autophagy as a novel mechanism.
				Limitation: Does not address the core issue of pharmacokinetic behavior in the disease state.

Table 10. Summary of cardioprotective studies on ASD VI

Reference	Study model	ASD VI Dose/Concentration	Key findings	Critical appraisal by this review
Li et al. (2010)	*In vitro*: H9c2 cardiomyocyte hypoxia/reoxygenation (H/R) injury	10, 20 μM (pre-treatment)	Activates the PI3K/AKT/CREB pathway, regulates Bcl-2/Bax, and inhibits apoptosis.	Strength: Provides a clear dose-response relationship and a classic anti-apoptotic mechanism.<br>Limitation: Simplified model lacking complexities of *in vivo* ischemia-reperfusion (e.g., inflammation).
Feng et al. (2020)	*In vitro*: H_2O_2-induced oxidative stress in H9c2 cells	25, 50, 100 μM	Alleviates oxidative stress and inhibits apoptosis by	Strength: Explores the protective mechanism under oxidative stress, a key factor in myocardial injury.

			activating the ATF6 pathway.	Limitation: Limited to cellular level; cardiac functional outcomes were not assessed.
Li et al. (2012)	*In vivo*: Myocardial ischemic injury animal model	30, 60, 120 mg/kg/d	Alleviates myocardial ischemic injury, improves cardiac function prognosis, and inhibits myocardial fibrosis.	Strength: Confirms therapeutic effects on functional and tissue remodeling outcomes *in vivo*.
				Limitation: Model type, animal number, and dosing regimen are not detailed, limiting reproducibility assessment.

Table 11. Summary of other pharmacological studies on ASD VI

Reference	Study model	ASD VI Dose/concentration	Key Findings	Critical appraisal by this review
Deng Yantao and Liu Yuan (2020)	*In vivo*: Rat model of Qi deficiency and blood stasis syndrome (TCM syndrome)	30, 120 mg/kg/d	Improves symptoms, regulates TNF-α, MDA, and SOD levels, showing promoting blood circulation effects.	Strength: Evaluated in a TCM syndrome-specific model, having certain characteristics.<br>Limitation: The standardization of the TCM syndrome model and the specificity of evaluation indexes are still debated.
Zhang et al. (2020)	*In vivo*: Mouse and rat thrombosis models	30, 120 mg/kg/d	Regulates the balance of 6-keto-PGF1α/TXB2 *in vivo* and inhibits platelet aggregation, showing	Limitation: Preliminary evidence. The study did not explore its selective effects on platelet aggregation

			anti-thrombotic effects.	induced by different agonists.
Zhou et al. (2012)	*In vitro*: U937 and HL-60 leukemia cells	10, 25, 50, 100 μM	Inhibits proliferation and induces apoptosis, related to down-regulation of Bcl-2, up-regulation of p53, and promotion of NO content.	Limitation: Very preliminary study limited to two hematological tumor cell lines. The mechanism exploration is superficial and lacks in-depth verification.
Wang Kun and He Benxiang (2022)	*In vivo*: Tendon injury animal model	10, 20, 40 mg/kg/d	Promotes tendon healing by normalizing abnormal ECM collagen metabolism and increasing TGF-β1 and PAI protein expression.	Limitation: Isolated evidence. The molecular mechanism description remains vague ('regulation of ECM metabolism'). Lacks follow-up studies.

Table 12 ASD VI multi-field research (bone/neurology/liver/cardiovascular) risk assessment Table

Study (First Author, Year)	A. Compounds & Materials	B. Experimental Design	C. Model Relevance	D. Data Analysis	E. Mechanism Depth	F. Limitation Discussion	Overall Risk Impression
**Osteoporosis research**							
Huang et al. (2018)	Low risk	Moderate risk	Low risk (BMSCs)	Low risk	Moderate risk (JNK pathway)	High risk	Moderate risk
Zhang et al. (2020)	Low risk	Moderate risk	Low risk (BMSCs, glucocorticoid model)	Low risk	Moderate risk (Multiple pathways)	High risk	Moderate risk
Wu et al. (2012)	Low risk	Moderate risk	Low risk (BMSCs)	Low risk	Moderate risk (Induced differentiation)	High risk	Moderate risk
**Neuroprotection research**							
Zhou et al. (2009)	Low risk	Moderate risk	Moderate risk (PC12 cells, Aβ injury)	Low risk	Moderate risk (Cell protection)	High risk	Moderate risk
Wang et al. (2023)	Low risk	Moderate risk	Moderate risk (sleep deprivation mice)	Low risk	Moderate risk (Neurogenesis)	High risk	Moderate risk
**Study on liver protection**							

Li et al. (2014)	Low risk	Moderate Risk	Low Risk (NAFLD mice)	Low risk	Moderate Risk (Lipid metabolism)	High Risk	Moderate risk
**Cardiovascular protection research**							
Feng et al. (2020)	Low risk	Moderate risk	Low risk (H9c2 myocardial cells)	Low risk	Moderate risk (ATF 6 pathway)	High risk	Moderate risk
**Anti-inflammatory/immune regulation research**							
Luo et al. (2023)	Low risk	Moderate risk	Low risk (Macrophages)	Low risk	Moderate risk (M1/M2 polarization)	High risk	Moderate risk
**Antithrombotic research**							
Zhang et al. (2020)	Low risk	Moderate risk	Low risk (Model of vein thrombosis in rats)	Low risk	Moderate risk (platelet aggregation)	High risk	Moderate risk
**Study on tendon repair**							
Wang Kun and He Benxiang	Low risk	Moderate risk	Moderate risk (Rabbit tendinopathy)	Low risk	Moderate risk (metabolism of ecm)	High risk	Moderate risk
**Anticancer research**							
Zhou et al. (2012)	Lowrisk	Moderate risk	Moderate risk (Leukemia cell lines)	Low risk	Moderate risk (Apoptosis-related)	High risk	Moderate risk
